# An ATP‐Driven N Protein–DDX21 Molecular Switch Dynamically Controls SARS‐CoV‐2 RNA G‐Quadruplex Heterogeneity

**DOI:** 10.1002/advs.77748

**Published:** 2026-09-16

**Authors:** Ya‐Ting Zheng, Li‐Yan Zhai, Jie Jin, Qiu‐Ying Chen, Hang Fu, Xue‐Qian Sun, Ying Lu, Hui Li, Xi‐Miao Hou

**Affiliations:** ^1^ College of Life Sciences Northwest A&F University Shaanxi China; ^2^ Beijing National Laboratory For Condensed Matter Physics Institute of Physics Chinese Academy of Sciences Beijing China; ^3^ University of Chinese Academy of Sciences Beijing China; ^4^ School of Systems Science and Institute of Nonequilibrium Systems Beijing Normal University Beijing China

**Keywords:** DEAD‐box helicases, G‐quadruplex, heterogeneity, nucleocapsid protein, SARS‐CoV‐2

## Abstract

The SARS‐CoV‐2 RNA genome functions as a highly structured regulatory scaffold. Although bioinformatic analyses predict widespread RNA G‐quadruplexes (G4s) across the viral genome, their structural diversity and regulatory mechanisms remain poorly understood. Here, we report a diverse landscape of viral G4s encompassing parallel and non‐canonical topologies with remarkable thermostability. Unlike typical eukaryotic G4s, these two‐tetrad viral G4s exhibit a hierarchical ion‐dependent mechanism, in which K^+^ establishes the core fold, and Mg^2^^+^ acts as a secondary regulator promoting conformational compaction. Single‐molecule FRET analysis further distinguishes rigid, long‐lived G4 folds from highly dynamic, metastable species, defining a continuum of conformational states along the viral genome. Functionally, we identify a synergistic yet competitive interplay between the viral nucleocapsid (N) protein and host helicase DDX21. While the N protein acts as a molecular chaperone to promote G4 folding, DDX21 selectively resolves these structures in an ATP‐dependent manner. Strikingly, N and DDX21 jointly constitute a finely tuned, ATP‐driven molecular switch, where ATP availability dictates the equilibrium between G4‐stabilized and resolved states. Our findings establish a mechanistic framework for the active regulation of SARS‐CoV‐2 RNA architecture and reveal a multilayered host–virus regulatory axis that modulates viral genome heterogeneity.

## Graphical abstract

An ATP‐driven molecular switch, comprising viral nucleocapsid (N) protein and host helicase DDX21, dynamically modulates SARS‐CoV‐2 RNA G‐quadruplex (G4) heterogeneity. These viral G4s feature non‐canonical ion‐dependence and act as energy‐sensitive structural checkpoints. This mechanism may toggle between G4 maintenance and resolution to orchestrate the viral life cycle in response to cellular ATP levels.

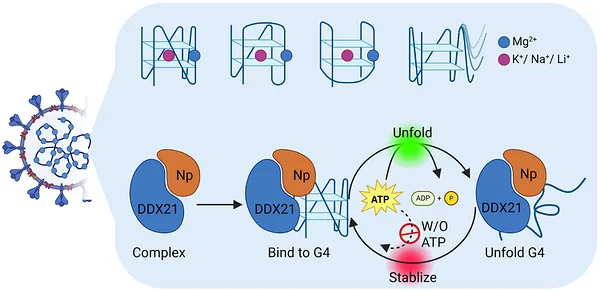

## 1 Introduction

The SARS‐CoV‐2 RNA genome functions as a highly organized regulatory scaffold that orchestrates the viral life cycle. Among the diverse RNA secondary structures embedded within viral genomes, G‐quadruplexes (G4s) are non‐canonical motifs formed by stacked guanine quartets stabilized by Hoogsteen hydrogen bonding and cation coordination [1]. Accumulating evidence indicates that RNA G4s serve as critical regulatory hubs in viral systems, influencing replication, transcription, translation, and genome packaging [2]. Computational analyses have consistently identified abundant putative G4‐forming sequences (PQSs) across the SARS‐CoV‐2 genome [3], with 14–25 conserved motifs on the positive strand alone [4, 5, 6]. Large‐scale evolutionary screening of >200 000 genomes has further revealed hundreds of PQSs and a small subset of ultra‐conserved elements (>98% conservation) [7, 8, 9, 10], including the well‐characterized RG‐1 motif within the nucleocapsid (N) gene [11]. The persistence of these motifs against the high mutation rate of RNA viruses strongly suggests that they are not incidental sequence features but functionally important genomic elements under selective pressure.

Consistent with this view, functional studies have demonstrated that viral RNA G4s are biologically active. Zhao et al. showed that RG‐1 forms a stable RNA G4 in cells and that G4‐stabilizing ligands suppress N protein expression by inhibiting translation [11]. Qin et al. further reported that G4‐targeting compounds reduce viral replication in cell culture and animal models, significantly lowering viral load and reducing lung pathology [12]. These findings establish SARS‐CoV‐2 RNA G4s as bona fide regulatory elements and compelling antiviral targets.

Despite these advances, the structural and dynamic properties of SARS‐CoV‐2 RNA G4s remain poorly defined. To date, no high‐resolution experimental structures are available, leaving the field largely reliant on in silico modeling. Hybrid quantum/classical simulations suggested that RG‐1 may adopt a two‐tetrad parallel G4 with a rigid core and flexible loops [13]. In contrast, more recent combined experimental and computational analyses proposed that RG‐1 predominantly forms a non‐canonical G‐triplex rather than a classical G4 under physiological conditions [14]. These disparate models suggest that SARS‐CoV‐2 RNA may access multiple canonical and non‐canonical G‐rich conformations. However, the structural heterogeneity across the viral genome, the equilibria between competing folds, and the real‐time conformational dynamics that govern these transitions remain largely unexplored.

Viral RNA structures do not operate in isolation but are dynamically remodeled by the host–virus proteome. The SARS‐CoV‐2 N protein is a multifunctional RNA‐binding protein that packages the viral genome [15], forms phase‐separated condensates with viral RNA [16], and scaffolds replication/transcription complexes [17]. Beyond its structural role, the N protein actively recruits host DEAD‐box helicases to viral replication sites [18, 19]. Proteomic studies have identified several DEAD‐box helicases, including DDX1, DDX3X, DDX5, and MOV10, as N‐protein interactors [20, 21, 22, 23], and notably, direct association with DDX21 has been observed [18]. Given that many of these helicases possess ATP‐dependent G4‐resolving activity [24, 25], we hypothesize that SARS‐CoV‐2 does not merely tolerate RNA G4s but employs a coordinated protein‐mediated machinery to dynamically “gate” their folding, ensuring genomic plasticity.

Here, we combine ensemble biophysical measurements with single‐molecule fluorescence imaging to address these questions. We demonstrate that SARS‐CoV‐2 RNA G4s are not static structural motifs but dynamic RNA elements that populate highly heterogeneous folding landscapes. We further uncover a non‐canonical Mg^2^^+^‐dependent stabilization mechanism in SARS‐CoV‐2 RNA G4s and resolve a broad kinetic spectrum ranging from rigid, long‐lived folds to highly metastable conformations. Moreover, we identify an ATP‐driven molecular switch orchestrated by the viral N protein and the host helicase DDX21, which cooperatively modulates G4 folding and accessibility. Together, these findings position viral RNA G4s as energy‐sensitive structural checkpoints governed by both intrinsic topology and coordinated host–virus interplay, highlighting new opportunities for antiviral intervention.

## 2 Results

### 2.1 SARS‐CoV‐2 Encodes Structurally Diverse and Thermodynamically Distinct RNA G4s

**FIGURE 1:**
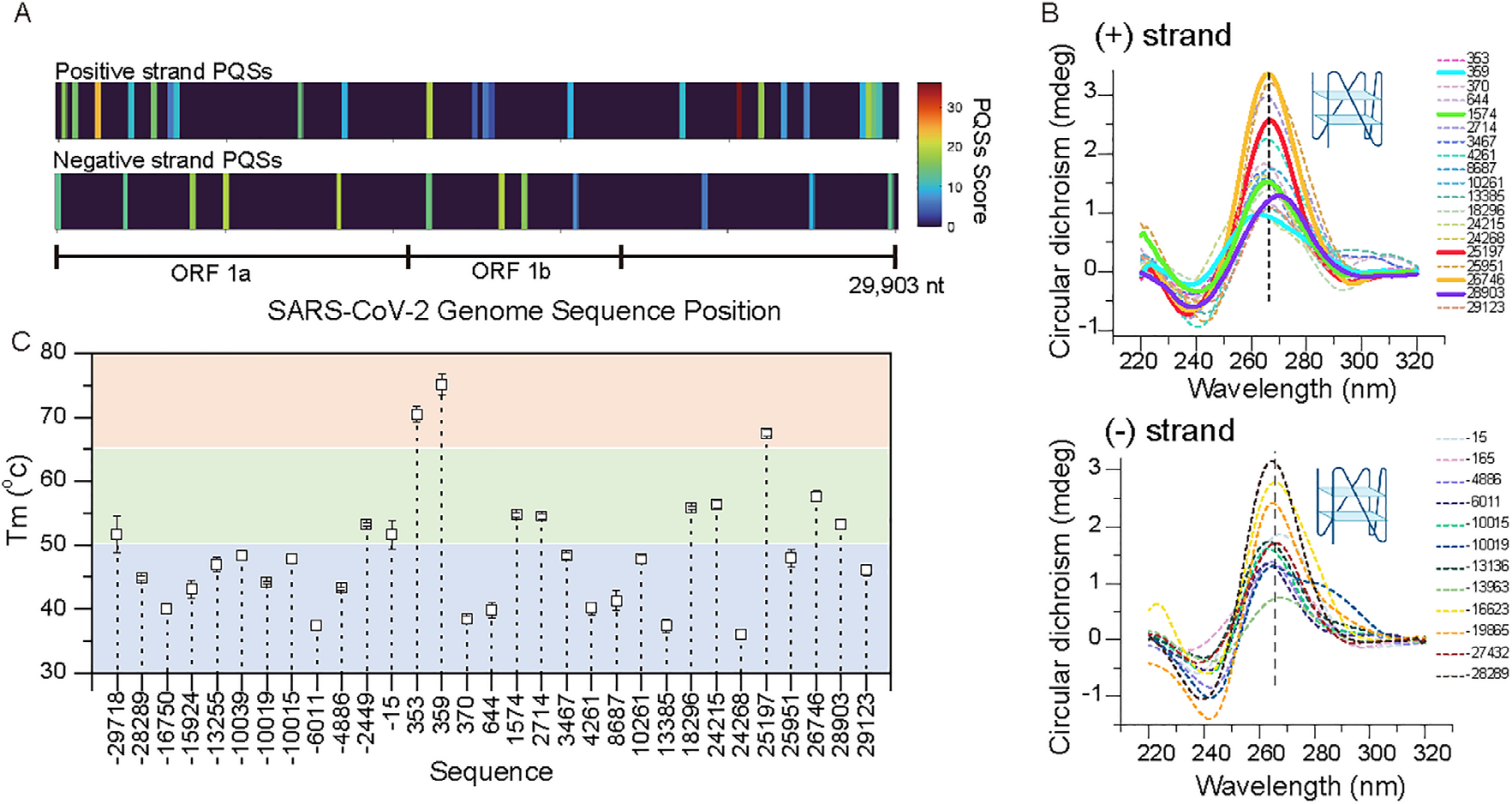
Formation and characterization of RNA G4s in the SARS‐CoV‐2 genome. (A) The heatmap of predicted PQSs located on the positive and negative strands of the SARS‐CoV‐2 genomic RNA. (B) The CD spectra of predicted PQSs, measured under physiological‐like conditions (20 mM Tris‐HCl, pH 7.5, 150 mM KCl). Sequences selected for further investigation are shown as bold lines. (C) Thermostability of these PQSs was assessed by CD melting. Regions with high (Tm>65°C), moderate (Tm = 50°C–65°C), or low thermal stability (Tm <50°C) are indicated by different colors.

To identify potential G4‐forming sequences within the SARS‐CoV‐2 genome, we utilized three independent bioinformatic tools, G4Hunter, PQSfinder, and QGRS Mapper, to scan the entire viral RNA across the positive and negative strands. This integrated analysis yielded 52 candidate PQSs (Table S1), among which 37 were unique and non‐overlapping after removing redundant predictions (Figure 1A and Figure S1A). Most PQSs conformed to a G_2_L_1_G_2_L_2_G_2_L_3_G_2_ motif, characteristic of two‐tetrad G4s, and displayed pronounced loop heterogeneity (0–19 nt) with asymmetric loop lengths (Figure S1B).

To experimentally validate G4 structure formation, we selected 31 representative, non‐identical PQSs for biophysical characterization (Table S2), based on the following criteria: (i) consensus support across G4Hunter, PQSfinder, and QGRS Mapper, (ii) grouping of overlapping predictions by genomic coordinates and selection of representative sequences, and (iii) representation of distinct genomic regions and loop‐length distributions to ensure coverage of structural diversity. Detailed selection criteria and scoring are provided in Data File S1.

CD spectroscopy under physiologically relevant conditions (20 mM Tris‐HCl, pH 7.5, 150 mM KCl) showed that 30 sequences adopted G4 structures (Figure 1B and Figure S2), exhibiting a characteristic positive peak near 265 nm and a negative band around 240 nm indicative of parallel topology, whereas several displayed additional signals near 295 nm consistent with hybrid conformations [26]. In contrast, most corresponding DNA sequences failed to form stable G4s under identical conditions (Figure S3). Fluorescence assays using the G4‐selective probes ThT and QUMA‐1 further corroborated G4 formation (Figures S4 and S5). One sequence (24215) lacked the expected ThT response and exhibited an atypical CD maximum at 258 nm, indicating a non‐G4 structure and resulting in 30 validated RNA G4s. Although parallel topology predominated, several sequences (644, 3467, 13385, and −10019) displayed additional CD features in the 282–303 nm range, suggesting non‐canonical or hybrid structures (Figure S2). This observation is consistent with previous combined CD and multiscale molecular modeling analyses showing that a SARS‐CoV‐2 RNA G4 can adopt both parallel and hybrid conformations [27]. Notably, the well‐characterized RG‐1 element (28903) exhibited a distinct CD maximum at 268 nm, underscoring the structural diversity of SARS‐CoV‐2 RNA G4s.

Thermodynamic profiling in Figure S6 further revealed a wide spectrum of G4 stabilities, which we categorized into three distinct groups (Figure 1C). The majority of sequences (60%) displayed low stability (Tm = 38°C–50°C), while a smaller group (30%) showed moderate stability (Tm = 50°C–65°C). Most strikingly, three sequences (353, 359, and 25197) exhibited exceptional thermal stability, with Tms exceeding 65°C. 359 even reached a high Tm of ∼76°C. NMR spectroscopy of this sequence (Figure S7) revealed imino proton peaks at 10.4–10.8 ppm, a hallmark of G4 hydrogen bonding [28], further confirming its G4 structure.

To investigate the thermodynamic basis underlying these stability differences, we performed van't Hoff analysis of CD‐melting data (Table S3). Beyond Tm measurements, this analysis decomposed G4 folding into enthalpic and entropic contributions and revealed substantial energetic heterogeneity among viral G4s. Thermodynamic parameters varied markedly across sequences (ΔH ≈ 100.4–266.5 kcal/mol; ΔS ≈ 0.32–0.83 kcal/(mol·K)), indicating distinct energetic origins of stabilization. Notably, highly stable G4s such as 353, 359, and 25197 also exhibited the highest unfolding free energies at physiological temperatures, indicating intrinsically greater stability. We next examined the relationships between loop length and thermodynamic parameters (Table S4). Total loop length showed moderate negative correlations with both ΔH (Spearman ρ = −0.589, permutation p = 0.0010) and ΔS (ρ = −0.571, permutation p = 0.0010). Among individual loops, only Loop 2 length showed significant negative correlations with ΔH (ρ = −0.504, p = 0.0032) and ΔS (ρ = −0.488, p = 0.0070), whereas Loop 1 and Loop 3 showed no significant associations. These results suggest that loop length, particularly Loop 2, is associated with the enthalpic and entropic contributions to SARS‐CoV‐2 RNA G4 stability.

To examine whether sequence length and composition contribute to the observed differences in Tm, we performed additional sequence‐level analyses of the 31 experimentally tested PQSs. Within the analyzed length range of 16–26 nt, full sequence length showed only a weak and non‐significant association with Tm (Spearman ρ = 0.195; Pearson r = 0.116), while individual loop lengths likewise showed no significant associations (Table S4), suggesting that sequence length alone was not a major determinant of Tm in this dataset. We next examined sequence‐dependent determinants of G4 stability using an analysis focused on loop composition (Tables S5 and S6). In contrast to overall sequence length, loop composition showed strong associations with Tm: total loop GC content was positively correlated with Tm (Spearman ρ = 0.823), whereas AU content was negatively correlated (ρ = −0.823). Loop C content and C‐minus‐U content were also positively correlated with Tm, suggesting that C‐enriched and U‐depleted loops are associated with greater G4 stability. Representative pairwise comparisons of overlapping or sequence‐similar PQSs further showed that differences in loop composition were associated with substantial differences in thermal stability (Table S7).

We used a two‐stage strategy to select sequences for detailed characterization. The initial set of 31 experimentally tested PQSs was selected to provide broad representation of the predicted SARS‐CoV‐2 G4 landscape, whereas a smaller subset was subsequently selected primarily based on the experimentally determined thermostability landscape. Because the biological functions of most individual SARS‐CoV‐2 G4s remain poorly understood, functional relevance could not be used as a systematic criterion for sequence selection. We selected representative G4s spanning distinct stability classes for more detailed characterization. In particular, 359 and 1574 were selected as complementary primary mechanistic models representing highly stable and moderately stable G4s, respectively, while additional sequences were included to extend the analysis across the stability range and provide independent biophysical validation (Table 1). This selection strategy allowed us to examine whether the observed effects extend across SARS‐CoV‐2 G4s with different intrinsic stabilities.

**TABLE 1:** Representative SARS‐CoV‐2 RNA G4s selected for detailed characterization.

Name	Sequence	Location	Thermostability	Role in this study	Rationale
359	GGAGACUCCGUGGAGGAGG	Nsp1	Highest	Primary mechanistic model	Selected as a highly stable viral RNA G4 for in‐depth investigation of ion‐, ligand‐, and protein‐mediated regulation.
1574	GGUGUUGUUGGAGAAGGUUCCGAAGG	Nsp2	Moderate	Primary mechanistic model	Selected as a representative moderately stable G4 with biophysical properties distinct from 359, enabling in‐depth comparative investigation.
28903	GGCUGGCAAUGGCGG	N protein	Moderate	Independent validation/Reference	Included based on previous cellular characterization [11, 12].
25197	GGCCAUGGUACAUUUGGCUAGG	Spike	High	Independent validation	Included as an independent high‐stability G4 to validate the Mg^2^^+^‐dependent stabilization.
26746	GGAUCACCGGUGGAAUUGCUAUCGCAAUGG	M protein	Moderate	Independent validation	Included as an additional moderately stable G4 to validate Mg^2^^+^‐dependent stabilization.
−28289	GGGGUGCAUUUCGCUGAUUUUGGGG	Negative strand	Low	Independent validation	Selected as a representative low‐stability G4 to extend ligand‐stabilization analysis.

To assess the ligand responsiveness of these G4s, we performed FRET‐melting assays on four representative sequences with high, moderate, and low Tm (359, 1574, 28903, and −28289) in the presence of six known G4 ligands. As shown in Figure S8, TMPyP4 and Phen‐DC3 exhibited significant stabilizing effects on all four G4s. 28903 even maintained its structure at 90°C in the presence of Phen‐DC3. These findings further confirm the formation of G4 structures and suggest that TMPyP4 and Phen‐DC3 possess potential antiviral activity.

Together, these findings identify SARS‐CoV‐2 RNA as a rich source of structurally diverse and thermodynamically distinct G4 elements. The exceptional stability of certain G4s, including 353 and 359 in nsp1 and 25197 in spike, highlights their potential for future functional and therapeutic investigation.

### 2.2 Unique Ion‐Dependence and Stabilization Mechanisms of SARS‐CoV‐2 RNA G4s

Canonical three‐tetrad G‐quadruplexes are typically stabilized by monovalent cations, particularly K^+^, which coordinate within the central channel of stacked G‐quartets. To determine whether SARS‐CoV‐2 RNA G4s follow this paradigm, we selected representative elements spanning distinct stability and biological contexts, including the highly stable 359‐G4, the moderately stable 1574‐G4, and 28903‐G4.

**FIGURE 2:**
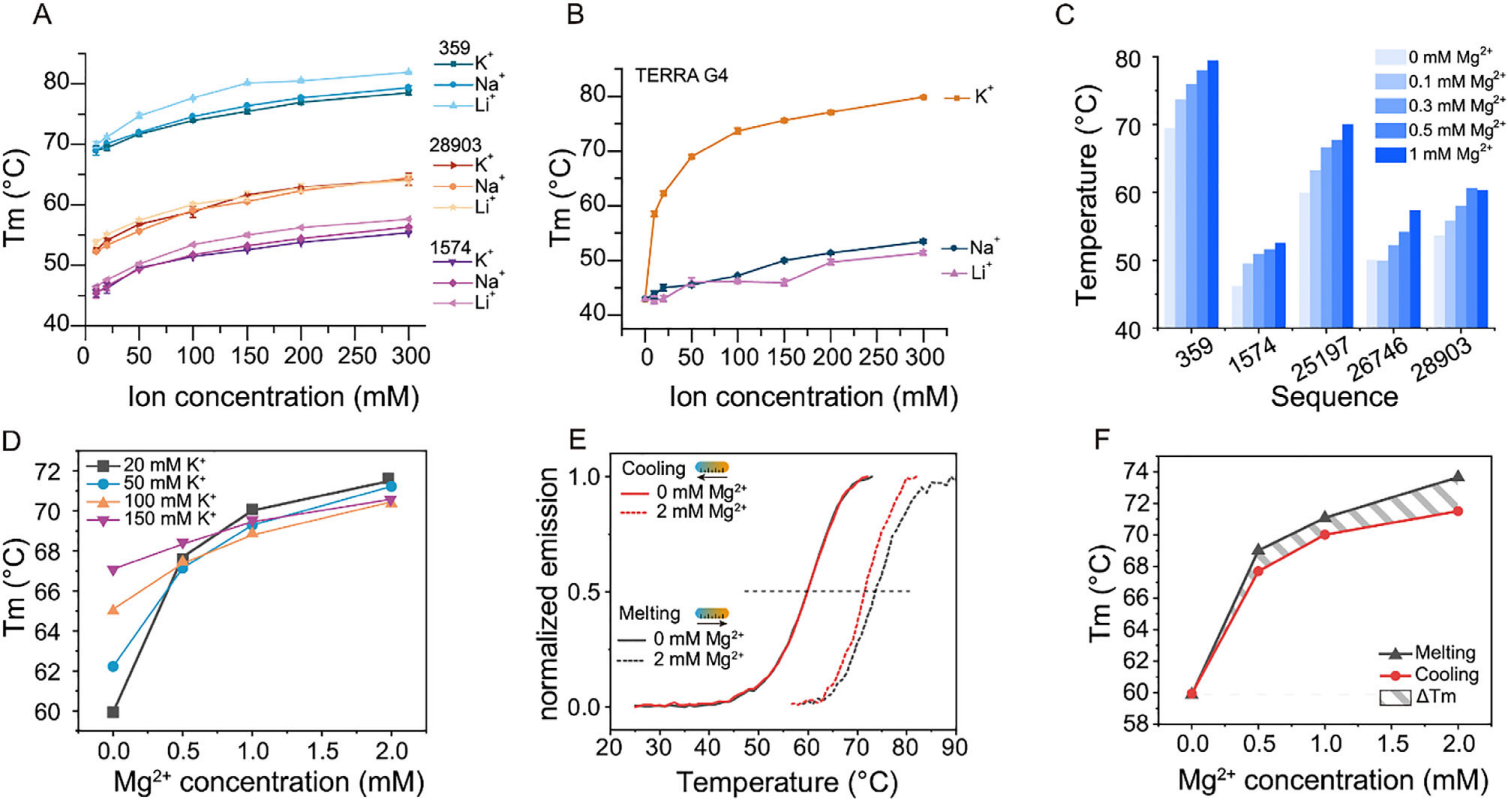
Monovalent and divalent cations differentially stabilize SARS‐CoV‐2 RNA G4s. (A) Tm of SARS‐CoV‐2 G4‐forming sequences 359, 1574, and 28903 in the presence of various types and concentrations of monovalent cations. (B) Tm of the canonical three‐tetrad TERRA G4 under the same conditions as a control. (C) Tm of 359, 1574, 25197, 26746, and 28903 in response to increasing [Mg^2^^+^] in a buffer containing 20 mM KCl. (D) 2D matrix scanning of 25197 stability under combinations of varying baseline [K^+^] (20, 50, 100, and 150 mM) and supplementary [Mg^2+^] (0–2 mM). (E) Cooling and melting profiles of 25197 in 20 mM K^+^ with 0 and 2 mM Mg^2+^. (F) Melting and cooling Tm values of 25197 as a function of [Mg^2+^], with thermal hysteresis values shown as ΔTm (defined as Tm, _melting_‐ Tm, _cooling_).

Across a broad range of monovalent cation concentrations (10–300 mM), SARS‐CoV‐2 RNA G4s exhibited only modest changes in Tm values (Figure 2A). This behavior contrasted sharply with that of the human telomeric RNA (TERRA) G4, whose stability was strongly enhanced by increasing K^+^ concentration (Figure 2B). Moreover, whereas TERRA G4s displayed pronounced ion specificity, SARS‐CoV‐2 G4s showed little discrimination among K^+^, Na^+^, and Li^+^. This lack of ion selectivity was further supported by CD‐melting profiles, which confirmed that the thermal stability of SARS‐CoV‐2 G4s remained largely unchanged regardless of the monovalent cation species present (Figure S9).

In contrast, divalent Mg^2^^+^ ions produced a pronounced, concentration‐dependent stabilizing effect on SARS‐CoV‐2 RNA G4s. Even at low millimolar concentrations (0.1–1 mM), Mg^2^^+^ substantially increased the melting temperatures of all tested viral G4s (Figure 2C). Two additional viral G4 sequences, 25197 (high Tm) and 26746 (moderate Tm), were included to validate this effect, both exhibiting increases in Tm of approximately 5°C–10°C. CD spectra further showed that Mg^2^^+^ did not induce detectable changes in G4 topology or overall folding (Figure S10), suggesting that Mg^2^^+^ stabilizes pre‐existing structures rather than promoting alternative conformations. To examine whether the divalent‐cation effect was specific to Mg^2^^+^, we further tested Ca^2^^+^, Mn^2^^+^, and Zn^2^^+^ under the same ionic conditions. Ca^2^^+^ produced a stabilizing effect similar to Mg^2^^+^ (Figure S11), whereas Mn^2^^+^ and Zn^2^^+^ produced non‐canonical fluorescence profiles without resolvable melting transitions, precluding reliable determination of Tm. These observations suggest that SARS‐CoV‐2 RNA G4s respond differently to different divalent cations.

To investigate the relationship between Mg^2^^+^‐mediated stabilization and basal K^+^ levels, we systematically examined 25197 under a matrix of K^+^ (20, 50, 100, and 150 mM) and Mg^2^^+^ (0–2 mM) concentrations. Mg^2^^+^ consistently enhanced G4 stability under all K^+^ conditions (Figure 2D), with the strongest effect observed at low K^+^ concentrations (20 mM), thereby mitigating the structural instability imposed by deficient monovalent cations. Interestingly, regardless of the initial K^+^ level, Mg^2^^+^ supplementation progressively shifted Tm values toward a similar upper range. These findings suggest a hierarchical stabilization model in which K^+^ establishes basal G4 stability, while Mg^2^^+^ provides an additional stabilizing contribution. As K^+^ concentration increases and the structure approaches maximal stability, the incremental effect of Mg^2^^+^ becomes progressively reduced.

To further examine the kinetic contribution of Mg^2^^+^, we compared cooling and melting profiles of 25197‐G4. In the absence of Mg^2^^+^, cooling and melting curves nearly overlapped, yielding negligible hysteresis (ΔTm ≈ 0), consistent with rapid and reversible folding transitions under near‐equilibrium conditions (Figure 2E). In contrast, increasing Mg^2^^+^ concentrations progressively increased the separation between cooling and melting curves, resulting in larger ΔTm values (Figure 2E,F). This thermal hysteresis indicates that Mg^2^^+^ not only enhances thermodynamic stability but also increases the kinetic barrier for structural transitions, suggesting that Mg^2^^+^ stabilizes viral G4s by reducing structural flexibility and slowing conformational rearrangements.

Overall, these findings reveal a distinctive cation‐dependent folding principle in SARS‐CoV‐2 RNA G4s and point to a mechanistically distinct mode of structural regulation within the viral genome.

### 2.3 Single‐Molecule FRET Reveals Structural Rigidity of 359

**FIGURE 3:**
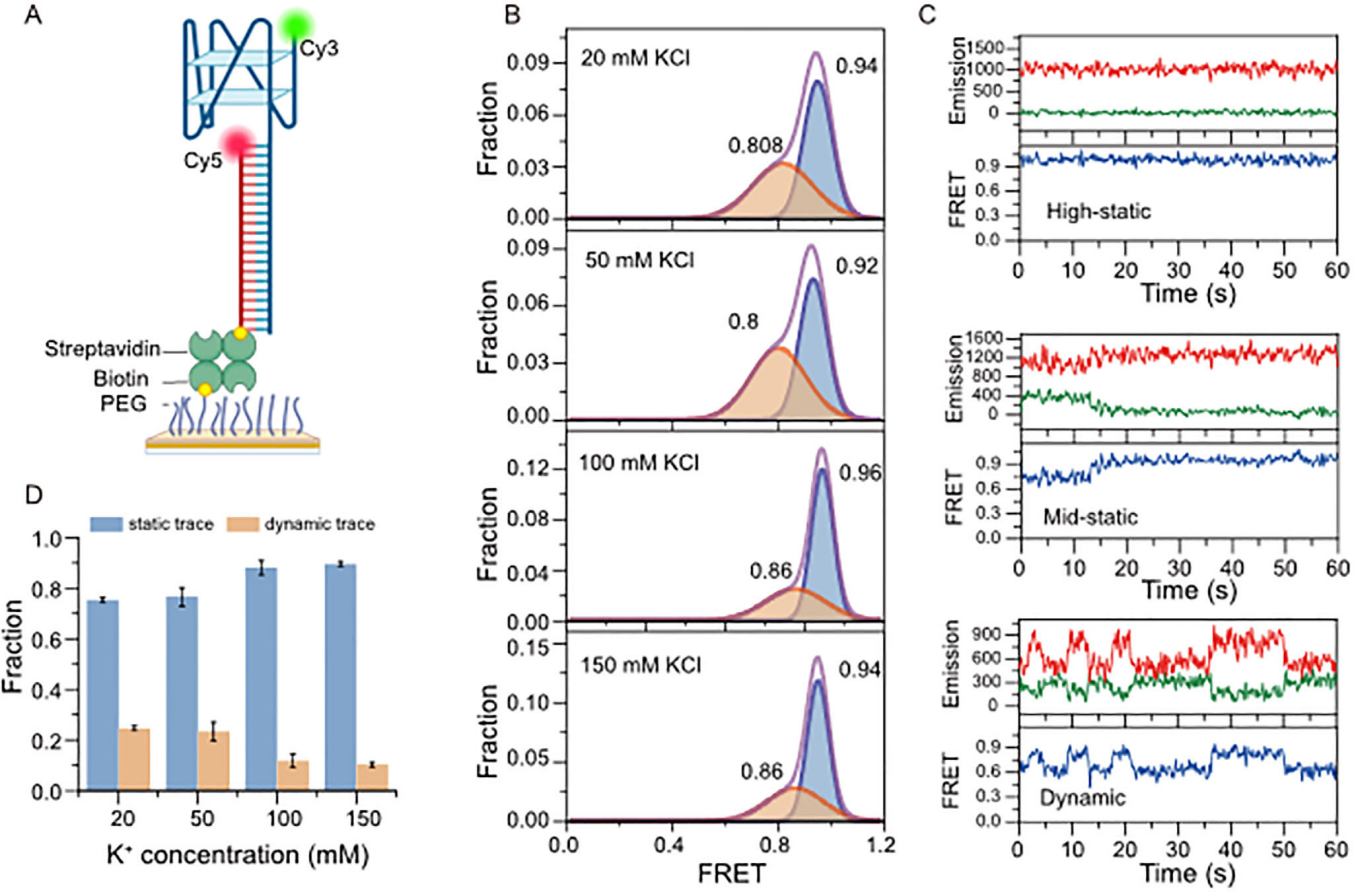
Single‐molecule FRET reveals high conformational stability of 359‐G4. (A) Schematic of the 359‐G4 construct. The G4 is flanked by Cy3 and Cy5 fluorophores at both ends and immobilized on the coverslip surface via biotin–streptavidin interaction. (B) FRET histograms of 359‐G4 at various [K^+^] in the absence of Mg^2+^, compiled from over 300 single‐molecule traces. (C) Representative fluorescence emission and corresponding FRET traces for high‐static, mid‐static, and dynamic molecules. (D) Proportions of static and dynamic FRET traces observed at different [K^+^].

To investigate the folding dynamics of SARS‐CoV‐2 G4s, we performed single‐molecule FRET measurements on two representative sequences: 359‐G4 (high stability) and 1574‐G4 (moderate stability). G4 structures were site‐specifically labeled with Cy3 and Cy5 fluorophores at the 3' end and the single‐to‐double‐strand junction, respectively (Figure 3A), allowing FRET efficiency to sensitively reflect their conformational states and changes [29].

Characterization of 359‐G4 folding under varying KCl concentrations (20–150 mM) revealed a bimodal FRET distribution with two peaks at ∼0.94 and ∼0.8–0.86 (Figure 3B). The higher‐FRET peak (0.94) was predominant across all conditions, and its relative population increased slightly with rising salt concentration, suggesting that elevated ionic strength promotes a more homogeneous and compact G4 conformation. Trajectory analysis classified smFRET traces into static and dynamic populations (Figure 3C). The static population was dominated by trajectories remaining in the high‐FRET state (∼0.94), while a small subset occasionally exhibited rare transitions between two states (1 transition per 60 s; referred to as mid‐static behavior). The fraction of dynamic traces decreased with increasing KCl concentration (Figure 3D), indicating that higher ionic strength stabilizes the G4 fold. At 150 mM KCl, approximately 90% of trajectories were static. These observations are consistent with the high thermostability of 359‐G4 observed in bulk measurements (Figure 1C).

To validate that the observed FRET dynamics originate from bona fide G4 folding, we performed multiple controls. The 4‐nt linker alone yielded a single stable high‐FRET population (∼0.95) across 20–150 mM KCl without dynamics, excluding contributions from duplex breathing (Figure S12A,B). In contrast, G‐to‐U substitutions (359Mut) broadened the FRET distribution and shifted it to lower values (Figure S12C), consistent with disruption of a compact G4 fold. A poly‐U control exhibited a distinct low‐FRET population peaked at ∼0.5, further excluding nonspecific structural compaction. Finally, the 4‐nt linker and 359‐G4 showed nearly identical high‐FRET states, excluding artifacts from the linker or labeling.

Together, these results confirm that the smFRET signals reflect intrinsic G4 folding. Under physiological monovalent salt conditions, 359‐G4 predominantly adopts a stable, structurally homogeneous fold with minimal conformational fluctuations.

### 2.4 1574 Displays Pronounced Structural Heterogeneity and Multiple Dynamic Folding Modes

**FIGURE 4:**
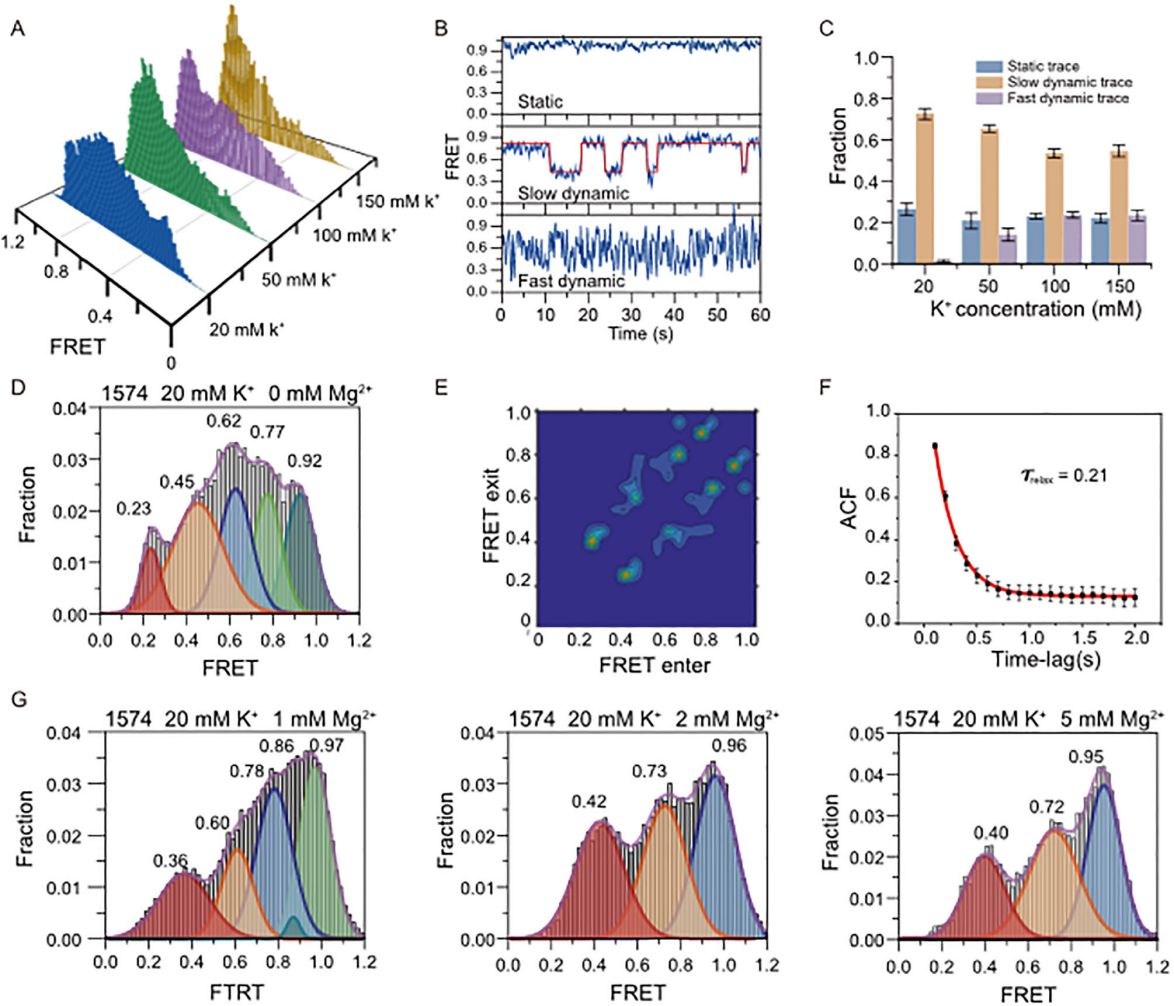
1574‐G4 shows structural heterogeneity with three distinct types of traces. (A) FRET histograms of 1574‐G4 under various [K^+^]. (B) Representative FRET traces illustrating the three distinct dynamic behaviors, with HMM idealization shown as a red trace overlaid on the slow dynamic trace. (C) Relative proportions of the three FRET trace types observed at different [K^+^]. (D) Gaussian fitting of the FRET histogram at 20 mM KCl resolved five distinct peaks. (E) Transition density plot (TDP) depicting FRET state transitions derived from the slow dynamic traces at 20 mM KCl. (F) Autocorrelation function (ACF) analysis of fast dynamic FRET traces at 20 mM KCl. The red curve represents a single‐exponential fit. (G) FRET histograms of 1574‐G4 measured in 20 mM KCl with increasing [Mg^2^^+^]. Each FRET histogram was compiled from >300 single molecules.

We next investigated the structural dynamics of 1574‐G4 across the same KCl gradient (20–150 mM). In sharp contrast to the rigid folding behavior observed for 359‐G4, smFRET analysis of 1574‐G4 revealed substantial structural heterogeneity across all salt conditions examined (Figure 4A). At low ionic strength (20 mM KCl), the FRET distribution was broad and multi‐peaked. Increasing KCl progressively shifted the ensemble toward higher FRET values, reflecting modest stabilization by K^+^, consistent with bulk FRET‐melting measurements (Figure 2A). Nevertheless, even at physiological salt concentrations (150 mM KCl), the FRET profile of 1574‐G4 remained markedly broader than that of 359‐G4, pointing to an intrinsically heterogeneous folding landscape.

Single‐molecule trajectories further revealed three distinct dynamic behaviors (Figure 4B and Figure S13A,C): a static population, slow stepwise interconversions between discrete FRET states, and rapid continuous fluctuations lacking well‐defined intermediates. For the slow dynamic molecules, hidden Markov modeling (HMM) idealization resolved stepwise transitions between discrete FRET levels, suggesting that these trajectories represent bona fide conformational interconversions rather than stochastic FRET noise (Figure 4B). The fast dynamic behavior was particularly notable, as it has not been commonly observed for canonical G4 structures, and likely reflects competition between G4 folding and alternative RNA secondary structures, such as transient stem–loops or partially folded intermediates [30, 31]. While the fraction of static molecules (∼25%) remained largely constant across salt conditions, increasing KCl shifted the dynamic population from slow interconversions toward fast, continuous fluctuations (Figure 4C).

At low salt (20 mM KCl), pooled trajectories revealed five discrete FRET states centered at 0.23, 0.45, 0.62, 0.77, and 0.92 (Figure 4D), suggesting the presence of at least five conformations. Transition density plot (TDP) analysis confirmed both the identities of these conformations and their interconversions (Figure 4E), supporting a highly dynamic and heterogeneous folding landscape. Dwell‐time distributions for individual FRET states derived from HMM‐idealized trajectories were shown in Figure S13B. Single‐exponential fits yield the corresponding dwell‐time constants (τ) ranging from 0.76 to 1.72 s.

To quantify the rapid fluctuation mode, autocorrelation function (ACF) analysis was performed on fast dynamic traces. The ACF decayed rapidly with a relaxation time of approximately 0.21 s, supporting sub‐second conformational exchange within the 1574‐G4 ensemble (Figure 4F). These findings are consistent with bulk thermal measurements showing intermediate stability for 1574‐G4 (Figure 1C).

### 2.5 Mg^2^^+^‐Induced Collapse of the Conformational Ensemble in 1574

To directly investigate the molecular basis underlying the Mg^2^^+^‐dependent stabilization observed in bulk measurements (Figure 2C), we next examined the conformational behavior of individual G4 molecules by smFRET (Figure 4G). 1574 was selected because its moderate stability and broad FRET distribution provide a wider conformational landscape and greater dynamic range for resolving Mg^2^^+^‐induced conformational redistribution. In the presence of 20 mM KCl, addition of Mg^2^^+^ up to 2 mM reduced the conformational multiplicity of 1574‐G4 to three dominant FRET states and effectively eliminated low‐FRET species. At higher Mg^2^^+^ concentrations (5 mM), the highest‐FRET state (∼0.95) became overwhelmingly predominant, indicating that Mg^2^^+^ strongly favors compact G4 conformations. At each concentration, three classes of traces could still be observed (Figure S14), suggesting that conformational dynamics persist but become increasingly restricted.

These observations provide a direct single‐molecule explanation for the bulk effects of Mg^2^^+^. Rather than inducing alternative G4 topologies, Mg^2^^+^ progressively collapses a heterogeneous conformational ensemble into a smaller population of compact states, providing a mechanistic basis for the increased thermal stability and hysteresis observed in bulk assays.

### 2.6 N Protein Preferentially Binds and Promotes Viral RNA G4s Folding

Viral RNA structures are shaped not only by their intrinsic folding propensities but also by viral and host RNA‐binding proteins (RBPs). Proteomic analyses have identified numerous RBPs associated with the SARS‐CoV‐2 genomic RNA during infection, among which the N protein is one of the most abundant viral factors [32].

To examine how RBPs engage SARS‐CoV‐2 G4‐forming regions, we performed fluorescence anisotropy assays [33] to measure the binding affinities of a selected panel of viral and host proteins toward the highly stable 359‐G4 under varying ionic conditions (Figure S15A). Among all proteins tested, N protein exhibited exceptionally strong and salt‐insensitive binding, with a dissociation constant (K_D_) of ∼10 nM at 20 mM KCl, and maintained high affinity up to 100 mM KCl. Broader substrate profiling revealed that N protein binds multiple DNA and RNA sequences but shows a clear preference for RNA G4s (Figure S15B). Size‐exclusion chromatography further indicated that G4 binding alters the oligomeric state of N protein, shifting it from a tetrameric (∼183 kDa) to a monomer‐like species (∼50 kDa) (Figure S15C–E), suggesting the formation of an N–G4 complex with a smaller apparent hydrodynamic size.

**FIGURE 5:**
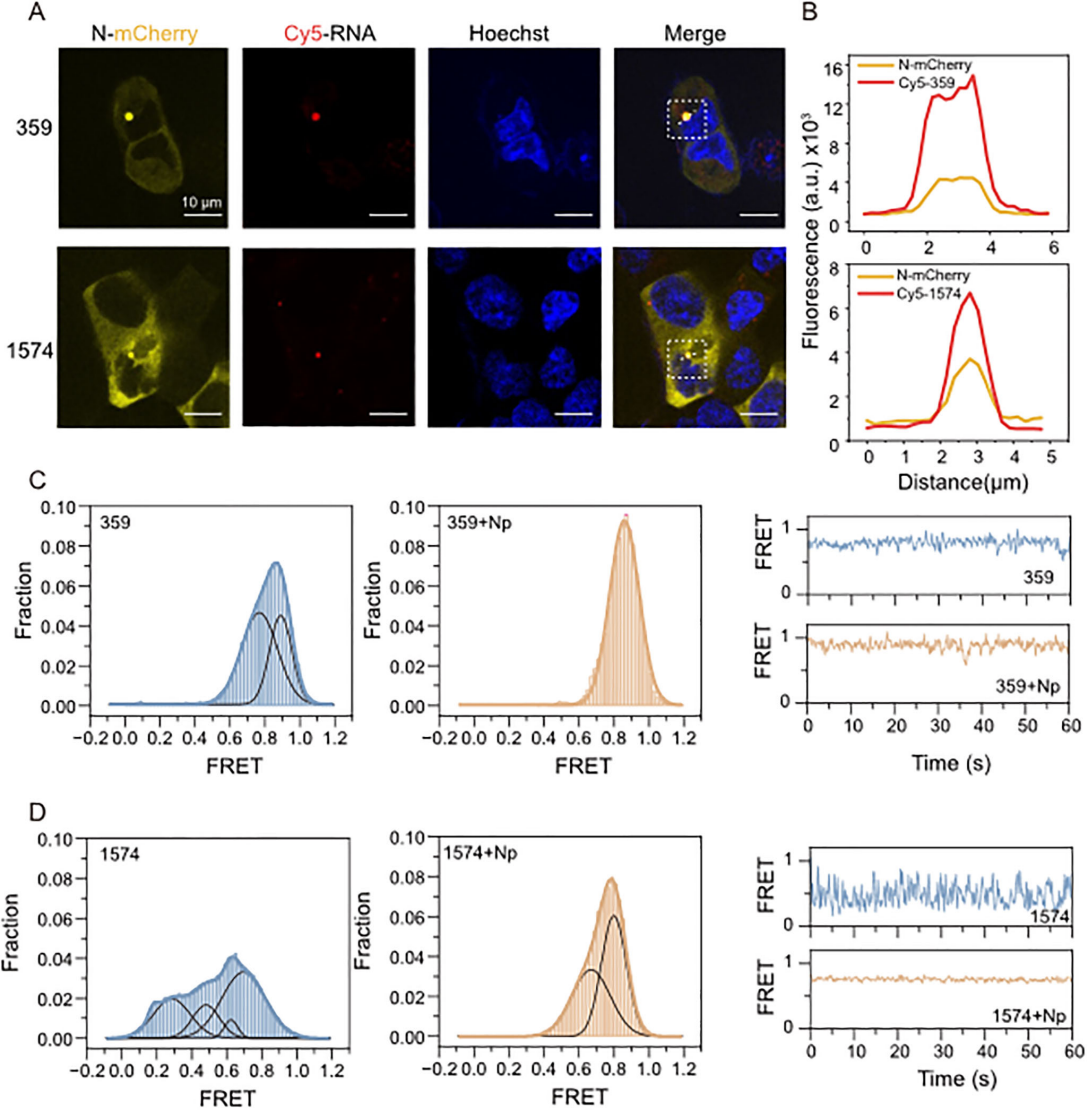
SARS‐CoV‐2 N protein (Np) preferentially binds to and stabilizes SARS‐CoV‐2 G4s. (A) Confocal fluorescence images showing HEK293T cells co‐transfected with N‐mCherry (yellow) and Cy5‐labeled 359‐G4 or 1574‐G4 (red). Nuclei were stained with Hoechst 33342 (blue). (B) Line‐scan profiles show co‐localization of N protein and G4 signals in cytoplasmic puncta. (C,D) FRET distributions and representative traces of 359 and 1574‐ G4s in the absence or presence of 100 nM N protein, measured in 20 mM Tris‐HCl (pH 7.5), 20 mM KCl.

Previous studies have demonstrated that SARS‐CoV‐2 PQSs such as 3467 and 28903 form G4 structures in cells, as detected by the G4‐specific BG4 antibody [11, 12]. To address the cellular relevance of N protein–viral G4 interactions, we performed fluorescence colocalization analysis in HEK293T cells using Cy5‐labeled viral RNA G4 and N‐mCherry. Confocal imaging revealed localized enrichment of N protein signals at cytoplasmic puncta formed by transfected viral RNA G4s (Figure 5A,B for 359 and 1574; Figure S16 for 28903), suggesting that N protein can access and spatially associate with G4‐containing viral RNA in a cellular environment. Single‐transfection controls for both components showed no detectable signal leakage between channels, ruling out spectral bleed‐through (Figure S16). Together, these observations provide supporting cellular evidence that viral RNA G4s are accessible by N protein in host cells.

To probe the structural consequences of N protein binding on viral G4s, we performed smFRET measurements on the 359 and 1574 in the reconstituted in vitro system. Addition of 100 nM N protein caused a substantial rightward shift accompanied by a marked narrowing of the FRET distributions for both molecules, indicating stabilization of the folded conformation (Figure 5C,D). Notably, the conformationally heterogeneous 1574‐G4 transitioned into a more uniform high‐FRET population (∼0.8). Consistent with this, smFRET trajectories showed markedly reduced conformational dynamics following N protein binding (Figure S15F). This behavior contrasts with the G4‐unfolding activities reported for certain viral nucleocapsid proteins, such as HIV‐1 NC [34], suggesting that SARS‐CoV‐2 N protein may actively promote or stabilize G4 folding as a chaperone rather than disrupt it.

To directly evaluate the binding strength of N protein to 359‐G4, we performed ligand competition assays using G4 ligands PhenDC3 and TMPyP4. Despite its high affinity for 359‐G4, PhenDC3 was efficiently displaced by N protein at concentrations below 500 nM, as indicated by anisotropy values reverting to the N‐bound level (∼250) (Figure S17A,B). Only at elevated PhenDC3 concentrations (1–2 µM) was partial resistance observed. Similarly, TMPyP4 was completely displaced by 100 nM N protein across all tested ligand concentrations (0–2 µM) (Figure S17C,D). These results highlight the robustness of the N–G4 interaction.

Altogether, the above findings underscore the central role of N protein in shaping viral RNA structure and reinforce its function as a primary G4 regulator.

### 2.7 Host DDX21 Helicase Resolves Viral G4s in an ATP‐Dependent Manner

RNA viruses, as intracellular parasites, frequently hijack host DEAD‐box helicases to facilitate genome replication and modulate host antiviral responses. Recent proteomic and immunoprecipitation‐based studies have consistently identified interactions between the SARS‐CoV‐2 N protein and multiple host RNA helicases [18, 35, 36, 37], suggesting that viral RNP complexes actively engage host RNA remodeling machinery during infection. Guided by their high affinity for 359‐G4 (Figure S15A), we focused on two helicases, DDX3X and DDX21, which have been reported to interact with RNA G4s [24, 25, 38] and are implicated in SARS‐CoV‐2 infection [18, 19, 21], to investigate their roles in viral G4 remodeling.

**FIGURE 6:**
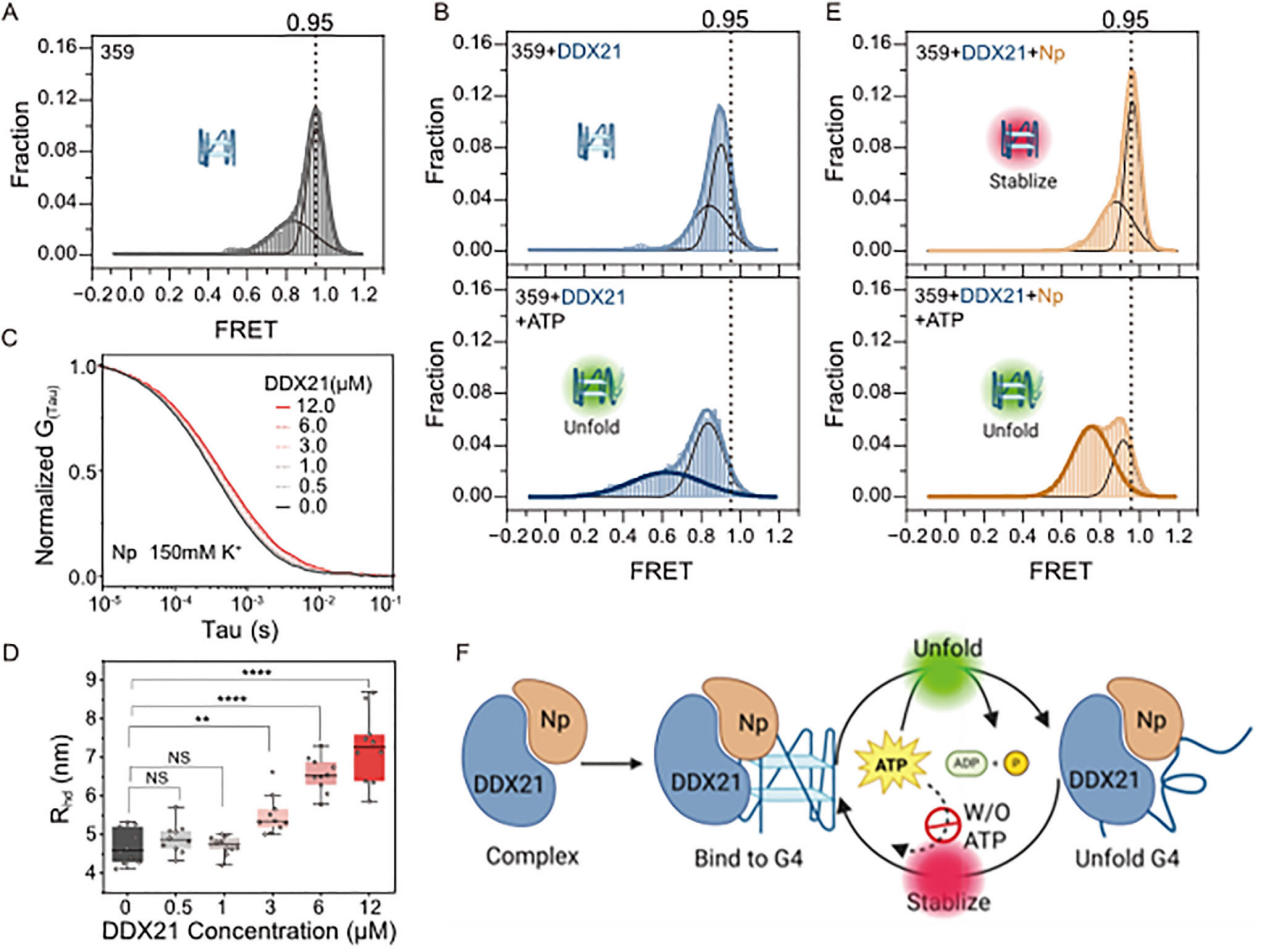
Antagonistic and cooperative remodeling of viral G4s by DDX21 and N protein. (A) FRET distributions of 359‐G4. (B) FRET distributions of 359‐G4 in the presence of 100 nM DDX21 without or with 2 mM ATP. (C) Normalized FCS autocorrelation curves of Atto488‐labeled N protein (20 nM) with increasing concentrations of DDX21 (0–12 µM) in the presence of 150 mM K^+^. (D) Hydrodynamic radius R_hd_ of N protein as a function of DDX21 concentration, derived from the FCS data (NS, not significant; ^**^, *p* < 0.01; ^****^, *p* < 0.0001). (E) FRET distributions of 359‐G4 in the presence of both 100 nM DDX21 and 100 nM N protein without or with 2 mM ATP. (F) Schematic model depicting the ATP‐driven N–DDX21 molecular switch that regulates SARS‐CoV‐2 G4 folding states.

Using single‐molecule FRET, we assessed their effects on two SARS‐CoV‐2 G4s: the stable 359‐G4 and the more transient 1574‐G4. DDX3X showed no detectable ability to disrupt the 359‐G4, even in the presence of ATP (Figure S18A). In contrast, DDX21 exhibited robust ATP‐dependent G4‐resolving activity (Figure 6A,B): while it had little effect without ATP, the addition of 2 mM ATP led to a marked shift of the FRET distribution toward lower values, indicating destabilization of the highly stable 359‐G4. Interestingly, DDX21 showed no significant impact on the 1574‐G4 (Figure S18B).

Additional binding assays showed that DDX21 binds both 359‐G4 and 1574‐G4 with high affinity (apparent K_D_ < 6 nM for both; Figure S18C), indicating that substrate recognition is unlikely to account for the observed selectivity. smFRET overlay analysis further revealed that DDX21 converts 359‐G4 into a lower‐FRET distribution that resembles the native conformational ensemble of 1574‐G4 (Figure S18D), suggesting that DDX21 does not completely unfold 359‐G4 into a single‐stranded state, but rather converts it into partially folded species.

Together, these results support a model in which DDX21 preferentially remodels stable, well‐folded G4 structures into dynamic partially folded ensembles, whereas substrates that already occupy such heterogeneous conformational space exhibit limited net responsiveness to DDX21.

### 2.8 An ATP‐Driven N–DDX21 Molecular Switch Regulates G4 Heterogeneity

The identification of N as a stabilizer and DDX21 as a resolver prompted us to investigate their functional interplay. Previous studies have provided substantial proteomic and cellular evidence supporting an interaction between the N protein and DDX21 [39, 40]. Large‐scale interactome analyses initially identified DDX21 as a major host interactor of N protein [35, 41], and subsequent cellular studies further validated the N–DDX21 association through reciprocal co‐immunoprecipitation experiments [18, 37], collectively supporting a functional interplay between these two proteins.

To further determine whether this interplay reflects direct physical association under defined solution conditions, we performed fluorescence correlation spectroscopy (FCS) [42]. In this assay, Atto488‐labeled N protein served as the fluorescent tracer, whereas DDX21 was titrated into the solution. FCS detects fluorescence fluctuations arising from the diffusion of the labeled N protein through the confocal volume. If DDX21 associates with N to form an N–DDX21 complex, the effective size of the fluorescent species is expected to increase, slowing the diffusion of fluorescent N and resulting in a shift of the autocorrelation curve and an increase in the apparent hydrodynamic radius (R_hd_).

Upon titration of DDX21 (0–12 µM) into a solution containing 20 nM Atto488‐labeled N protein under physiological salt conditions (150 mM KCl), a clear dose‐dependent rightward shift of the autocorrelation curves was observed (Figure 6C). Correspondingly, the derived R_hd_ increased from ∼4.5 to ∼7.0 nm (Figure 6D), providing direct evidence for a physical interaction between N protein and DDX21 in solution. Importantly, pre‐incubation of N protein with a 10‐fold excess of unlabeled 359‐G4 did not affect the subsequent FCS profiles (Figure S19), suggesting that the interaction is unlikely to arise from RNA‐mediated bridging.

To mechanistically dissect how N‐DDX21 association modulates viral G4 conformational dynamics, we reconstituted the system in vitro and performed single‐molecule FRET analysis. Under ATP‐free conditions, the N protein–DDX21 complex exhibited a G4‐stabilizing effect identical to that of N protein alone on both 359‐G4 (Figure 6E, upper panel) and 1574‐G4 (Figure S18E, left panel), indicating that the effect of N protein predominates in the absence of ATP. In contrast, ATP shifted the system into a G4‐destabilizing mode on the highly stable 359‐G4 (Figure 6E, lower panel), indicating that DDX21‐mediated helicase activity becomes predominant upon ATP activation. On the intrinsically less stable 1574‐G4 substrate, only minimal unfolding was observed (Figure S18E, right panel), similar to DDX21 alone (Figure S18B).

We next asked whether this ATP‐driven switch represents a general property of the N–DDX21 interplay or depends on RNA structural context. Using duplex RNA as a control, we found that N protein promoted RNA strand annealing (Figure S20A,B), whereas DDX21 exhibited ATP‐dependent unwinding and ATP‐independent annealing activities (Figure S20C,D). Notably, when both proteins were present, N protein overrode DDX21‐mediated unwinding even under ATP‐rich conditions (Figure S20E,F), shifting the reaction toward a net annealing outcome (Figure S20G). This contrasts sharply with the behavior on G4 substrates, where ATP enables DDX21 to overcome N‐mediated stabilization.

Together, these findings indicate that the N–DDX21 interplay functions not as a universal RNA‐remodeling module but as a G4‐specialized regulatory switch, in which ATP availability dynamically shifts the balance between N protein‐mediated stabilization and DDX21‐driven resolution (Figure 6F).

## 3 Discussion

**FIGURE 7:**
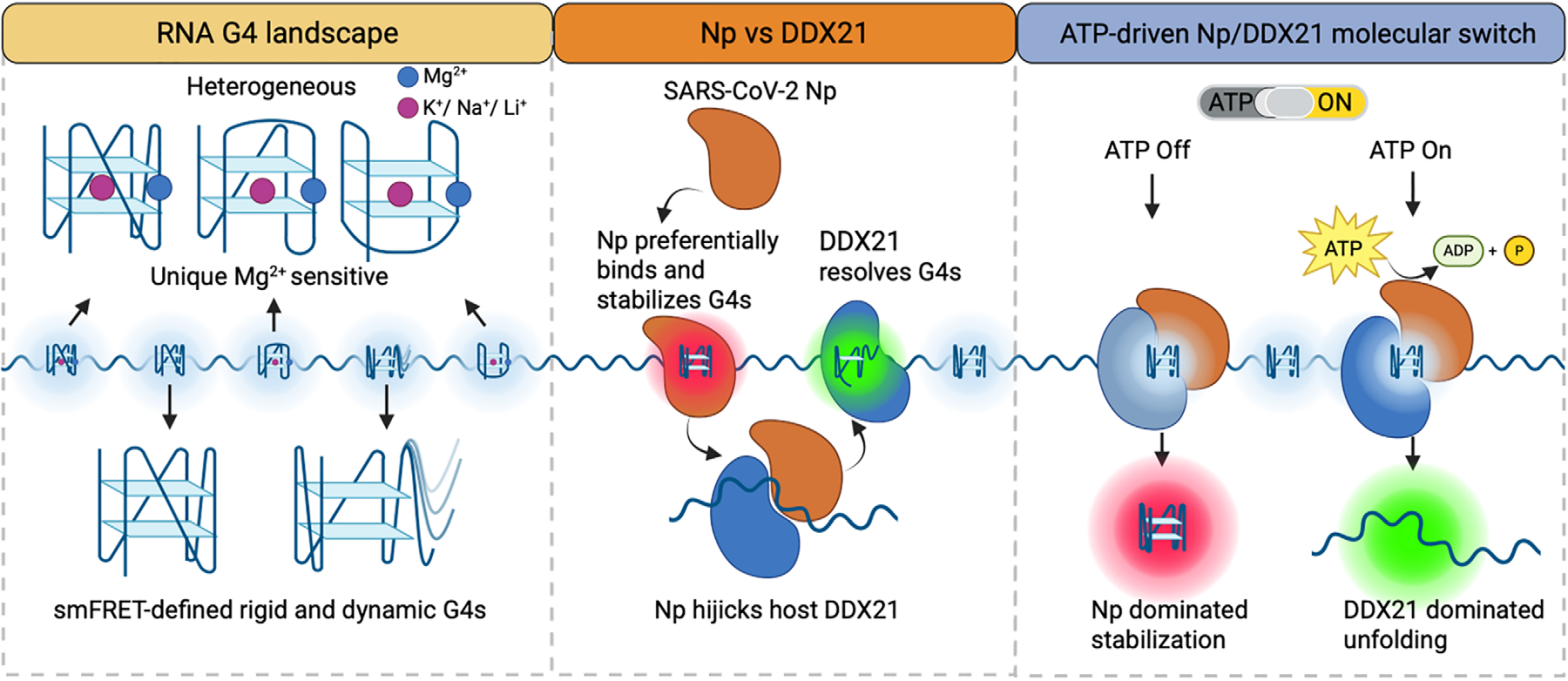
ATP‐dependent molecular switch controlling the conformational landscape of SARS‐CoV‐2 G4s.

In this study, we reveal a previously unrecognized layer of structural heterogeneity within the SARS‐CoV‐2 RNA genome. Using single‐molecule FRET, we have further mapped a dynamic folding landscape that distinguishes rigid, persistent scaffolds from metastable, rapidly interconverting species. Beyond these intrinsic properties, we identify a sophisticated regulatory interface where the viral N protein and host helicase DDX21 jointly constitute an ATP‐driven molecular switch to precisely tune G4 folding states. Overall, our findings establish a framework in which viral RNA architecture is not fixed but dynamically tuned by both sequence context and regulatory factors (Figure 7).

### 3.1 Conservation and Structural Diversity of Coronavirus G4s

G4‐forming sequences are not unique to SARS‐CoV‐2 but represent a recurrent feature of human coronavirus genomes [2]. Comparative bioinformatic analyses have identified putative G4‐forming sequences across all seven human coronaviruses (HCoV‐229E, HCoV‐NL63, HCoV‐OC43, HCoV‐HKU1, SARS‐CoV, MERS‐CoV, and SARS‐CoV‐2), with conserved loci particularly evident among SARS‐related coronaviruses [4]. This observation may suggest that, despite the rapid sequence evolution that generally characterizes RNA virus genomes, at least a subset of G4‐forming regions may be maintained under evolutionary constraints. Subsequent systematic biophysical analyses not only confirmed the conservation of these sequences from SARS‐CoV, SARS‐CoV‐2, and MERS‐CoV, but also demonstrated that these elements can adopt diverse structural states, including monomolecular and multimolecular G4s and, in some cases, equilibria between G4 and hairpin conformations [43]. Functionally, these G4s have been shown to regulate viral protein expression and replication [11, 12]. Building on these insights, we performed a systematic biophysical survey of 31 SARS‐CoV‐2 G4‐forming sequences, enabling a comprehensive analysis of their conformational landscape and stability determinants. Our study further extends these comparative observations by resolving distinct and interconverting conformational states of SARS‐CoV‐2 G4s at the single‐molecule level and by demonstrating their regulation by viral and host proteins.

### 3.2 Dynamic Coexistence of G4s and Alternative RNA Structures

While molecular dynamics simulations often provide static snapshots of G4 folds [13, 27], our CD and smFRET data provide direct evidence that these PQS elements occupy a heterogeneous conformational landscape rather than adopting single, static folds.

For 359‐G4, the dominant high‐FRET population corresponds to a compact G4 state, whereas a persistent minor population at lower FRET was also observed (Figure 3B). This species may represent a partially folded intermediate, potentially analogous to G‐triplex‐like states reported for 28903 [14]. Supporting this interpretation, increasing [K^+^] progressively reduced this minor population while favoring the mature G4 state. In contrast, 1574‐G4 exhibited multiple FRET populations and rapid state transitions, indicating a dynamically interconverting ensemble. Such rapid fluctuations are uncommon for canonical G4 systems and instead suggest structural heterogeneity involving alternative RNA conformations. RNA secondary structure prediction further indicates that local stem–loop conformation may form and compete with G4 folding (Figure S21A). In addition, non‐canonical features observed in CD spectra, including peak shifts detected for 28903 (Figure S2), may also reflect coexistence of alternative structural states such as G‐triplex‐like species. Together, these observations support a model in which SARS‐CoV‐2 RNA G4s populate a dynamic equilibrium involving fully folded G4s, partially folded intermediates, and alternative RNA conformations such as stem‐loops.

The biological relevance of this heterogeneity is further highlighted by the genomic context of these sequences. Previous experimental and computational studies have shown that many PQSs in the SARS‐CoV‐2 genome are embedded within larger stem–loop architectures [30, 31, 44, 45]; for example, 359 has been reported to reside within a 46‐nt stem–loop (SL7) (Figure S21B). Our finding that the same sequence can independently adopt a highly stable G4 conformation suggests that these RNA elements may access multiple structural states depending on environmental conditions. In this framework, stem–loop structures could represent kinetically accessible states, whereas G4 conformations may correspond to more compact and stabilized structures favored under specific conditions. External regulatory factors, including Mg^2^^+^, the N protein, and host helicases, may further modulate this balance, enabling the same class of RNA element to function either as a stable structural scaffold (e.g., 359) or as a highly responsive structural module (e.g., 1574) under different cellular or viral environments.

### 3.3 Possible Mechanisms Underlying Cation‐Dependent Stabilization of SARS‐CoV‐2 RNA G4s

A striking feature of SARS‐CoV‐2 G4s is their non‐canonical response to the ionic environment. In three‐tetrad eukaryotic G4s, stabilization typically requires coordination of two monovalent cations within the central channel, where they directly bind between stacked G‐quartets to stabilize the core [46]. The binding of the first cation is rapid, while association of the second often becomes rate‐limiting [46, 47]. In contrast, two‐tetrad G4s, which predominate in the SARS‐CoV‐2 genome, require only a single central cation, making their folding less sensitive to monovalent ion concentration [46].

While monovalent ions establish the basal structural framework, Mg^2+^ acts as a critical regulator (Figure 2C,D), elevating Tm toward a convergent thermodynamic ceiling across varying K^+^ conditions. Under low [K^+^], Mg^2^^+^ exerts a strong stabilizing influence, whereas at high [K^+^], its impact is markedly diminished. Such behavior suggests that Mg^2^^+^ does not simply add to K^+^‐dependent stability but instead functions to saturate the conformational folding space. Previous structural analysis has identified Mg^2^^+^ coordination sites outside the G‐quadruplex central channel, involving phosphate groups within loop/groove regions [48]. The Mg^2^^+^‐dependent stabilization observed here may therefore arise, at least in part, from electrostatic interactions with the phosphate backbone, potentially contributing to charge screening and reduced flexibility in the loop/groove regions.

This thermodynamic model is directly supported by our smFRET data (Figure 4G), where Mg^2+^ titration simplifies the heterogeneous conformational landscape by eliminating flexible, low‐FRET species and driving a “collapse” of the ensemble into dominant, highly compact folds. Mechanistically, this Mg^2^^+^‐induced restriction of conformational freedom is expected to reduce conformational entropy and increase the effective energy barrier for structural transitions, consistent with the pronounced increase in cooling–melting thermal hysteresis (Figure 2F).

Collectively, these findings challenge the prevailing view that G4 stability is governed primarily by monovalent ions [49], and instead suggest that SARS‐CoV‐2 RNA G4s exploit a hierarchical ion‐dependent stabilization mechanism in which Mg^2^^+^ serves as a critical electrostatic and dynamic regulator. Whether this Mg^2^^+^‐dependent response represents a broader property of RNA G4s or is particularly pronounced in viral G4s remains to be determined. Such divalent‐cation sensitivity may be physiologically relevant in cellular environments, where local ionic conditions fluctuate during infection and may bias viral RNA folding pathways toward G4 formation.

### 3.4 Viral G4s may Serve as Energy‐Sensitive Structural Checkpoints

Our findings suggest that viral and host proteins play important roles in shaping SARS‐CoV‐2 G4s (Figure 7). The N protein binds RNA G4s with nanomolar affinity and robustly stabilizes folded conformations, effectively suppressing intrinsic dynamics and shifting heterogeneous G4 populations toward more uniform, compact states. Given the role of viral RNA in driving liquid‐liquid phase separation (LLPS) of the N protein [50], these stabilized G4s may serve as structural scaffolds that facilitate the organization of viral ribonucleoprotein complexes within condensates, potentially safeguarding the viral genome from premature degradation or detection by host innate immune sensors.

Conversely, host DDX21, which has also been reported to partition into phase‐separated compartments [51], functions as an ATP‐dependent counter‐regulator that actively resolves these structures. The functional interplay between N protein and DDX21 may generate a regulatory system whose output is influenced by cellular energy status. Under ATP‐limiting conditions—often associated with cellular stress or late‐stage infection—N‐mediated stabilization may predominate, potentially acting as a “braking” mechanism that transiently restrains replication‐associated processes and preserves genomic integrity under adverse conditions. In contrast, when ATP is abundant, DDX21's remodeling activity prevails, resolving G4 “roadblocks” to license rapid viral RNA synthesis and translation.

Therefore, we propose that SARS‐CoV‐2 RNA G4s may function as energy‐sensitive structural checkpoints that couple viral RNA metabolism to the host cell's metabolic state. One plausible mechanism involves N protein recruiting DDX21 to specific G4 loci, forming a molecular switch that awaits an ATP signal to trigger (Figure 7). This framework provides a new dimension to our understanding of how viruses exploit host factors to dynamically tune their own genomic architecture. As both N protein and DDX21 are capable of phase separation, their potential co‐condensation may provide localized environments that enrich viral RNA and facilitate DDX21 recruitment, thereby modulating the efficiency of G4 remodeling. The functional implications of phase separation in this regulatory framework await future experimental validation.

## Author Contributions

**Ya‐Ting Zheng**: methodology, investigation, formal analysis, writing – original draft, Writing – review and editing, data curation. **Li‐Yan Zhai**: methodology, data curation, investigation, writing – original draft, writing – review and editing, formal analysis. **Jie Jin**: investigation. **Qiu‐Ying Chen**: investigation. **Hang Fu**: investigation. **Xue‐Qian Sun**: investigation. **Ying Lu**: methodology, software. **Hui Li**: methodology, software. **Xi‐Miao Hou**: conceptualization, supervision, funding acquisition, project administration, writing – original draft, writing – review and editing.

## Conflicts of Interest

The authors declare no conflicts of interest.

## Supporting information




**Supporting File 1**: advs77748‐sup‐0001‐SuppMat.docx.

**Supporting File 2**: advs77748‐sup‐0002‐SuppMat.xlsx.

## Data Availability

The data that support the findings of this study are available on request from the corresponding author. The data are not publicly available due to privacy or ethical restrictions.
