# Supplementary material for: An ATP‐Driven N Protein–DDX21 Molecular Switch Dynamically Controls SARS‐CoV‐2 RNA G‐Quadruplex Heterogeneity
Source: Adv Sci (Weinh). 2026 Sep 16:e77748. Online ahead of print. doi: 10.1002/advs.77748 (PMC13583083; doi:10.1002/advs.77748)
Supplement: Supplementary file 1 [file ADVS-9999-e77748-s001.docx]

**Supporting Information**

**An ATP-Driven N Protein–DDX21 Molecular Switch Dynamically Controls SARS-CoV-2 RNA G-Quadruplex Heterogeneity**

**Table of contents**

Materials and Methods………………………………………………………………………2

Prediction of PQSs in SARS-CoV-2 genome…………………………………………..2

Loop sequence comparison and stability association analysis………………………2

RNA oligonucleotides and sample preparation.…………………………….…………3

Protein expression and purification………………….………………………………….3

N Protein Fluorescent Labelling………………………………………………………...4

Circular dichroism (CD) spectroscopy and melting analysis…………………………4

FRET melting………………………………………………………….……………….….4

Thioflavin T (ThT) and QUMA-1 Fluorescence assays………………..………….…..5

1D ¹H Nuclear Magnetic Resonance (NMR)………………..……………..…….……..5

Fluorescence anisotropy binding assays………………………………...……………..5

Single-Molecule FRET (smFRET)……………………………………………………….5

Size exclusion chromatographic analysis of N–G4 complexes……………….……...6

Construction of pcDNA3.1(+)-N-mCherry Plasmid…………………………………….6

Cell Culture and Transfection…………………………………………………………….6

Cellular colocalization imaging…………………………………………………………...7

Fluorescence correlation spectroscopy (FCS) protein-protein interaction analysis**.**…………7

Stopped-Flow FRET Assay…………………………………………………………….…7

Supporting Tables……………………………………………..……………………………...8

Supporting Figures………………………………………….……………………………….18

**Materials and Methods**

**Prediction of PQSs in SARS-CoV-2 genome.** PQSs in the SARS-CoV-2 genome (NC_045512.2) were identified using a combination of three independent algorithms: G4Hunter, PQSfinder, and QGRS Mapper. The complete genomic RNA sequence, including both positive and negative strands, was analyzed to ensure comprehensive coverage. G4Hunter was run with a sliding window size of 25 nt and a score threshold of 1.0, optimized for two-tetrad G4 detection. PQSfinder settings were adjusted to capture sequences with a window size of 30 nt, a threshold of 10, and loop sizes between 0 and 17 nt. QGRS Mapper was used with default parameters for a window size of 30 nt, G≥2, and loop size 0-17 nt.

Candidate PQSs conforming to the two-tetrad G4 motif G_2_L_1_G_2_L_2_G_2_L_3_G_2_ were compiled from the three prediction outputs. To evaluate algorithmic support, a consensus score was assigned to each candidate according to the number of prediction criteria fulfilled: QGRS Mapper score ≥13, PQSfinder score ≥15, and |G4Hunter score| ≥1.0. Thus, each sequence received a consensus score ranging from 0 to 3. Overlapping candidates were grouped according to their genomic coordinates, and representative sequences were selected by considering consensus score, predicted G4 core, loop length distribution, strand origin, genomic location, and suitability for oligonucleotide synthesis and experimental validation. For highly overlapping candidates, the sequence with stronger algorithmic support or a more compact G4 core was generally retained; however, nested or boundary-shifted sequences were also retained when needed to represent boundary diversity within a G-rich locus.

In addition to algorithm-predicted PQSs, one rationally designed derivative sequence (-10019) was included for experimental validation. This sequence was derived from the highly overlapping -10015/-10039 G-rich region and was used as an intermediate boundary construct to improve representation of this G-rich locus in the experimental panel. The experimentally tested 4261 sequence represents a one-nucleotide boundary-shifted construct corresponding to the predicted 4262 candidate. The consensus scoring, genomic annotation, overlap analysis, and sequence selection rationale for all candidate PQSs are provided in Supplementary Data File 1, which serves as a curated decision matrix documenting the filtering and prioritization process.

Based on the combined strategy, 31 representative PQSs were selected from the candidate panel for subsequent CD, CD-melting, fluorescent probe, and NMR analyses. The selected panel covered both positive- and negative-strand candidates, different genomic regions including coding sequences and untranslated regions, and diverse loop-length arrangements, thereby enabling systematic evaluation of sequence-dependent RNA G4 formation and stability.

**Loop sequence comparison and stability association analysis.** The 31 experimentally tested PQS sequences listed in Table S2 were used for the loop sequence comparison and stability association analysis. For each selected sequence, the three loop regions were extracted according to the predicted G₂-loop1-G₂-loop2-G₂-loop3-G₂ motif. The loop sequences, loop lengths, nucleotide counts, and nucleotide percentages were calculated for each candidate. The three loops were also concatenated to calculate combined loop features, including total loop GC content, AU content, C content, U content, C-minus-U percentage, and Shannon entropy. GC, AU, C, and U contents were calculated as (G+C)/L, (A+U)/L, C/L, and U/L, respectively, where L is the total length of the concatenated loop sequence. C-minus-U content was calculated as C/L - U/L. Shannon entropy was calculated as H = -Σb pb log2(pb), where pb is the frequency of base b among A, C, G, and U.

Experimentally measured Tm values were matched to the corresponding sequence identifiers. Candidates were classified as high stability (Tm > 65 °C), moderate stability (50 °C ≤ Tm ≤ 65 °C), or low stability (Tm < 50 °C). Group-level summaries were calculated by averaging loop features within each stability class.

Associations between loop features and G4 thermal stability were evaluated using both Spearman rank correlation and Pearson correlation. For each loop feature, sequences with valid feature and Tm values were included in the correlation analysis. Spearman correlation was used to assess monotonic relationships between loop features and Tm without assuming a linear relationship, whereas Pearson correlation was used to evaluate linear associations. For Pearson correlation analysis, each loop feature was denoted as X, and the corresponding Tm value was denoted as Y. The Pearson correlation coefficient r was calculated as:

r = Σi(Xi - X̄)(Yi - Ȳ) / [√Σi(Xi - X̄)² √Σi(Yi - Ȳ)²]

For Spearman rank correlation analysis, X and Y were first converted into ranks, R(Xi) and R(Yi), and Spearman ρ was calculated using the same Pearson correlation formula on the ranked values. For each loop feature, sequences with valid feature and Tm values were included in the correlation analysis.

Two-sided empirical p values were estimated using 5,000 permutations. In each permutation, Tm values were randomly reassigned among candidates while the loop features were kept fixed, and the correlation coefficient was recalculated. The empirical p value was defined as the proportion of permutations producing an absolute correlation coefficient equal to or greater than the observed value, and was calculated as:

p = [N(|rperm| ≥ |robs|) + 1] / (B + 1)

where robs is the observed correlation coefficient, rperm is the correlation coefficient obtained after permutation, and B = 5000. The same permutation procedure was applied to both Pearson r and Spearman ρ.

Pairwise comparisons were further performed for overlapping or sequence-similar candidates. Candidate pairs were retained if they showed genomic interval overlap, normalized sequence identity ≥ 0.55, or edit distance ≤ 8 nt. For each retained pair, Tm differences, sequence identity, loop-length differences, and loop nucleotide composition differences were compared.

**RNA oligonucleotides and sample preparation.** RNA oligonucleotides were synthesized and HPLC-purified by GenScript Biotech (Nanjing, China). For ensemble and single-molecule experiments, RNAs were either unlabeled or site-specifically labeled with 5′-FAM/3′-TAMRA or Cy3/Cy5 fluorophore pairs, as indicated. RNAs were resuspended in RNase-free water and quantified using a NanoDrop spectrophotometer. For G4 folding, RNA samples were diluted to 4 µM in folding buffer (20 mM Tris–HCl, pH 7.5, 20–150 mM KCl), heated to 95 °C for 5 min, and slowly cooled to room temperature over 7 h to promote G4 formation. DNA oligonucleotides were synthesized by Sangon Biotech (Shanghai, China) and treated similarly.

**Protein expression and purification.**

*N protein.* The recombinant full-length N protein gene was cloned into the pET15b-SUMO vector and expressed in *E. coli* BL21(DE3). Cultures were grown at 37 °C in LB medium to an O_D600_~0.6, induced with 0.3 mM IPTG, and incubated at 18 °C for 16 h. Cells were harvested and lysed by sonication in a lysis buffer consisting of 20 mM Tris-HCl (pH 7.5), 0.5 M NaCl, and 12% (v/v) glycerol. The clarified lysate was loaded onto a Ni-NTA affinity column, washed with lysis buffer containing 10 mM imidazole, and eluted with elution buffer [20 mM Tris-HCl (pH 7.5), 0.5 M NaCl, 12% glycerol, and 350 mM imidazole]. Following ULP1 protease cleavage and a reverse Ni-NTA step to remove the 6×His-SUMO tag, N protein was further purified via a HiTrap SP cation-exchange column using a linear gradient of 0 to 1.0 M NaCl in a buffer containing 20 mM Tris-HCl (pH 7.5), 1.5 mM DTT, 2 mM EDTA, and 12% glycerol.

*DDX3X helicase.* The DDX3X gene was cloned into the pET15b-SUMO vector and transformed into *E. coli* BL21(DE3). Cultures were grown at 37 °C to an O_D600_~0.65. Expression was induced with 0.3 mM IPTG at 18 °C for 15 h. Cells were harvested and lysed by sonication in 20 mM Tris-HCl (pH 7.5), 0.5 M NaCl, and 12% glycerol. The clarified lysate was purified by Ni-NTA affinity chromatography, washed with lysis buffer containing 10 mM imidazole, and eluted with elution buffer containing 20 mM Tris-HCl (pH 7.5), 0.5 M NaCl, 12% glycerol, and 100 mM imidazole. Following ULP1 protease cleavage and a reverse Ni-NTA step to remove the 6×His-SUMO tag, the digested target protein sample was loaded onto a HiTrap SP cation-exchange column and eluted with a linear gradient of 0 to 1.0 M NaCl in 20 mM Tris-HCl (pH 7.5), 1.5 mM DTT, 2 mM EDTA, and 12% glycerol.

*DDX21 helicase.* The DDX21 gene was cloned into the pET15b-SUMO vector and transformed into *E. coli* BL21(DE3). Cells were cultured at 37 °C to an O_D600_~0.6, induced with 0.2 mM IPTG, and incubated at 18 °C for 16 h. After cell harvesting and sonication, the clarified lysate was subjected to primary Ni-NTA affinity chromatography and eluted with elution buffer [20 mM Tris-HCl (pH 7.5), 0.5 M NaCl, 12% glycerol, and 100 mM imidazole]. The 6×His-SUMO tag was removed by ULP1 protease cleavage coupled with overnight dialysis and a reverse Ni-NTA pass. The target protein was then loaded onto a Heparin affinity column in buffer (25 mM HEPES, pH 7.0, 2 mM EDTA, 1 mM DTT, 100 mM NaCl, and 20% glycerol) and eluted with a linear NaCl gradient (0 to 1.0 M). Finally, DDX21 was polished by size-exclusion chromatography on a Superdex 200 Increase 10/300 GL column.

All finalized protein samples were concentrated using centrifugal filters, snap-frozen in liquid nitrogen, and stored as single-use aliquots at −80 °C.

**N Protein Fluorescent Labeling.** Purified full-length N protein was first buffer-exchanged into an amine-free conjugation buffer (0.1 M NaHCO_3_, pH 8.3) to prevent interference with the coupling reaction. The protein was then conjugated with Atto488 NHS ester (Leica Microsystems, AD48835) by mixing the dye and protein at a dye-to-protein molar ratio of 1:1. The mixture was incubated for 6 h at room temperature with gentle agitation, strictly protected from light. Excess unreacted dye was efficiently removed by size-exclusion chromatography on a Superdex 200 Increase 10/300 GL column. The labeled protein was concentrated using centrifugal filters and snap-frozen in liquid nitrogen and stored as single-use aliquots at −80 °C until use.

**Circular dichroism (CD) spectroscopy and melting analysis.** CD spectra were recorded on a Jasco J-1500 spectropolarimeter using a 1 mm path length quartz cuvette. Pre-folded RNA samples (4 µM) in 20 mM Tris–HCl, pH 7.5, 20-150 mM KCl, and 0-2 mM MgCl_2_ were scanned from 220–320 nm at 25°C. For melting analysis, CD signals were monitored as temperature increased from 25°C to 95°C at 1°C/min. The melting temperatures (Tm) and van’t Hoff enthalpy values used for thermodynamic analysis were obtained by global fitting of the multiwavelength temperature-scan data using the Global3 software package (Applied Photophysics). Thermodynamic parameters were calculated according to the van't Hoff relationship, ΔG = ΔH - TΔS. At the melting temperature, ΔG = 0, and therefore ΔS = ΔH/Tm (with Tm expressed in Kelvin). The free energy at a given temperature was then calculated as ΔG(T) = ΔH - TΔS; accordingly, ΔG(25°C) = ΔH - 298.15ΔS and ΔG(37°C) = ΔH - 310.15ΔS. To assess ion dependence, CD spectra and melting curves were also obtained in buffers containing different monovalent (KCl, NaCl, LiCl) at indicated concentrations.

**FRET melting.** Dual-labeled RNA sequences (5'-FAM and 3'-TAMRA) were diluted to 0.5 µM in G4 folding buffer (20 mM Tris-HCl, pH 7.5, containing 20–150 mM KCl and 0–2 mM MgCl_2_). To induce G4 folding, the RNA solutions were heated to 95°C for 5 minutes and then gradually cooled to room temperature overnight. For general Tm determination, the FRET melting assay was performed by increasing the temperature from 25°C to 95°C at a ramp rate of 1°C/min. Mg²⁺ concentrations were limited to 2 mM to ensure reliable determination of Tm, as higher concentrations excessively stabilized certain sequences, resulting in incomplete melting profiles that precluded accurate Tm determination. To specifically explore the effects of Mg^2+^ on G4 folding kinetics, a separate thermal cycling experiment was conducted on selected RNA sequence under varied Mg^2+^ concentrations. For this specific sequence, consecutive cooling and melting profiles were recorded: the temperature was first decreased from 95°C to 25°C and subsequently increased from 25°C to 95°C, both in 1°C/min increments. Throughout all measurements, the donor fluorescence intensity was recorded at each step using a Bio-Rad CFX96 system. All measurements were performed in triplicate, and Tm values were derived from the midpoint of each transition. For the kinetic assays, ΔTm (defined as Tm,_melting_ − Tm,c_ooling_) was calculated accordingly.

**Thioflavin T (ThT) and QUMA-1 Fluorescence Assays.** RNA G4 formation was monitored using ThT and QUMA-1 fluorescence assays. Reactions were performed at 25 °C in a buffer containing 150 mM KCl, 0.5 µM ThT or QUMA-1, and 0–1 µM pre-annealed RNA. Fluorescence emission spectra were recorded on a BioTek Synergy H1 plate reader, with excitation at 425 nm (ThT) and 555 nm (QUMA-1), and emission ranges of 450–718 nm (ThT) and 600–800 nm (QUMA-1). Fluorescence intensity was plotted as a function of RNA concentration.

**1D ¹H Nuclear Magnetic Resonance (NMR).** 1D ¹H NMR spectra were acquired at 298 K using a Bruker 600 MHz spectrometer. RNA samples (200 µM, 500 µL) were dissolved in 90% H₂O/10% D₂O buffer containing 20 mM K-phosphate (pH 7.0) and 150 mM KCl. The samples were then annealed by heating in boiling water for 5 minutes, followed by slow cooling to room temperature in an insulated container to promote RNA secondary structure formation. Spectra were typically acquired with a minimum of 3600 scans. The imino proton region (10.5–12 ppm) was analyzed to identify characteristic G4 signals.

**Fluorescence anisotropy binding assays.** Binding affinity of proteins to RNA G4s was assessed by fluorescence anisotropy using 5'-FAM-labeled RNA (5 nM) and serial dilutions of proteins (0–300 nM) in 20 mM Tris-HCl pH 7.5, 20, 50, or 100 mM KCl. Measurements were performed on Infinite F200 (TECAN). K_D_ values were calculated by fitting the binding curves to a single-site binding model using the Hill equation. For N protein–ligand competition assays, 5 nM pre-folded 359 G4 was incubated with fixed concentrations of ligand (0–2 μM) at 37°C for 30 minutes in 20 mM Tris-HCl (pH 7.5) and 20 mM KCl. Subsequently, gradient concentrations of N protein (0–500 nM) were added, and fluorescence anisotropy was measured under the same buffer conditions.

**Single-Molecule FRET (smFRET).** The smFRET experiments were conducted using a home-built total internal reflection fluorescence (TIRF) microscope. RNA G4 constructs were internally labeled with a FRET donor (Cy3) and acceptor (Cy5) via modified nucleotides and annealed by heating to 95 °C followed by gradual cooling in a folding buffer containing 20 mM Tris-HCl (pH 7.5), 20–150 mM K⁺, and variable Mg²⁺ concentrations as indicated. 100 pM dual-labeled RNAs were immobilized via biotin-streptavidin on PEGylated quartz slides. FRET trajectories were collected in imaging buffer (20 mM Tris-HCl, pH 7.5, 20–150 mM KCl) supplemented with an oxygen scavenger system (0.8% D-glucose, 1 mg/mL glucose oxidase (266,600 units/mg), 0.4 mg/mL catalase (2,000–5,000 units/mg), and 1 mM Trolox). To examine the effects of proteins, the imaging buffer was further supplemented with 100 nM N protein, 100 nM DDX21, 100 nM DDX3X, or 100 nM N protein plus 100 nM DDX21, with or without 2 mM ATP/2 mM MgCl₂, as indicated in the corresponding experiments. The laser power was set to 60 mW, and videos were recorded with an exposure time of 100 ms for 60 seconds. FRET efficiency was calculated as the ratio of acceptor intensity to the sum of donor and acceptor intensities. FRET efficiency histograms were compiled from at least 300 molecules per condition and fitted with single or multi-peak Gaussian distributions.

Following compilation of FRET efficiency histograms, individual trajectories were smoothed using a 7-frame median filter and classified through a two-step procedure. First, trajectories were categorized as static or dynamic based on fluctuation amplitude. For each trajectory, the fluctuation amplitude (amp90–10) was calculated as the difference between the 90th and 10th percentiles of the FRET values. Trajectories were defined as dynamic if amp90–10 exceeded 0.20, or if they exhibited a sustained state transition characterized by a FRET change ≥0.20 maintained for ≥7 consecutive frames. All other trajectories were classified as static.

Dynamic trajectories were subsequently classified into slow and fast regimes according to their dynamical behavior. This hierarchical classification reflects the intrinsic differences in conformational complexity while maintaining a consistent physical basis for distinguishing discrete-state kinetics from continuous fluctuations across different constructs. For 1574-G4, trajectories that could be robustly segmented into discrete states using hidden Markov modeling (HMM), with stable state assignments and convergent dwell-time distributions across independent runs, were classified as slow dynamic trajectories, consistent with discrete-state conformational interconversion. In contrast, trajectories that failed to yield stable state assignments, reproducible state discretization, or consistent dwell-time distributions under HMM analysis were classified as fast dynamic trajectories, reflecting continuous or highly heterogeneous fluctuations not amenable to discrete-state modeling. Fast dynamic behavior was not observed for 359-G4 under the same analytical framework, consistent with its predominantly discrete-state or static conformational behavior.

Slow dynamic trajectories were analyzed using hidden Markov modeling (HMM) implemented in HaMMy software, consistent with their discrete-state behavior. Dwell-time histograms for each FRET state were fitted with a single-exponential decay, N(t) = Aexp(-t/τ), to determine the apparent leaving rate, k = 1/τ.

Fast dynamic trajectories, which did not support discrete-state idealization under HMM analysis, were analyzed using autocorrelation function (ACF) analysis. FRET fluctuations were calculated as δE(t) = E(t) - <E>, and the normalized ACF was calculated as C(Δt) = <δE(t)δE(t+Δt)>/<δE(t)^2>. The resulting C(Δt) was fitted with a single-exponential function, C(Δt) = Aexp(-Δt/τ_relax_) + C0, to extract the apparent relaxation time τ_relax_ characterizing the fast dynamics.

**Size exclusion chromatographic analysis of N–G4 complexes.** Purified N protein (40 µM) was incubated with pre-folded 7T-111-7T G4 with increasing concentrations (100-1200 µM) in binding buffer (20 mM Tris-HCl, pH 7.5, 150 mM KCl, 1 mM DTT) for 30 minutes at room temperature. The mixture (50 µL) was loaded onto a Superdex 200 Increase 10/300 GL column (Cytiva) pre-equilibrated with the same buffer and eluted at a flow rate of 0.5 mL/min using ÄKTA pure system. Elution profiles were monitored by UV absorbance at 280 nm and 260 nm. The column was calibrated using a set of protein standards with molecular weights (MW) of 13.7, 44.3, 150, and 670 kDa. A linear regression was established by plotting the logarithm of the MW (logMW) as a function of the elution volume (*Ve*). The apparent MW of the N–G4 complexes was subsequently determined by interpolating their observed *Ve* values into this standard calibration curve.

**Construction of** **pcDNA3.1(+)-N-mCherry Plasmid.** The target plasmid was constructed by PCR-based cloning. Briefly, the vector backbone pcDNA3.1(+)-N-3×Flag and mCherry insert were amplified from the corresponding template plasmids using high-fidelity DNA polymerase. PCR products were purified by agarose gel extraction and assembled using an OK Clon DNA assembly kit according to the manufacturer’s instructions. The assembled product was transformed into competent *E. coli* cells and plated on ampicillin-containing LB agar plates. Positive colonies were screened by colony PCR and confirmed by Sanger sequencing.

**Cell Culture and Transfection.** The human embryonic kidney cell line HEK293T was used. Cells were maintained in Dulbecco’s modified Eagle’s medium (DMEM) supplemented with 10% fetal bovine serum (FBS, GIBCO) and 1% penicillin-streptomycin (GIBCO). The cells were cultured at 37 °C in a humidified incubator containing 5% CO_2_. For transfection experiments, HEK293T cells were seeded into 35-mm confocal dishes the day before transfection. When the cells reached approximately 50-60% confluence, pcDNA3.1(+)-N-mCherry (500 ng/μL) and Cy5-labeled RNA oligonucleotides (50 nM) were sequentially transfected using Lipofectamine^TM^ 3000 (Invitrogen). The medium was replaced with fresh complete medium approximately 24 h post-transfection, and cells were observed by microscopy approximately 48 h post-transfection.

**Cellular colocalization imaging.** Fluorescence images were obtained using a spinning disk confocal microscopy system based on a microscope (Olympus IX73) equipped with a Yokogawa W1 spinning-disk unit. Images were captured with a 60× oil-immersed objective (1.50 N.A., Olympus) and a back-illuminated EMCCD camera (Andor iXon Life 888). The pixel size is 0.2166 µm. For multichannel imaging, three distinct lasers were utilized for imaging different probes: a 561-nm laser for mCherry; a 637-nm laser for Cy5 fluorophores; and a 405-nm laser for Hoechst 33342. To minimize spectral bleed-through, images were acquired sequentially by automatically switching the corresponding emission filters using the CSU motorized filter wheel: a B447/60 bandpass filter for Hoechst 33342, a B617/73 bandpass filter for mCherry, and a B685/40 bandpass filter for Cy5. For all three channels, the exposure time was maintained at 100 ms with an EM gain of 200.

**Fluorescence correlation spectroscopy (FCS) protein-protein interaction analysis.** FCS was used to examine potential interactions between N protein and DDX21 in vitro using a commercial bench-top system (CorTector™ SX200, LightEdge Technologies Limited, China). Atto488-labeled N protein was prepared as described in the N protein fluorescent labeling section.

For binding measurements, 20 nM labeled N protein was incubated with increasing concentrations of DDX21 (0–12 μM) in a buffer containing 20 mM Tris-HCl (pH 7.5) and 150 mM KCl for 10 min in a total volume of 20 μL. Prior to data acquisition, the effective detection volume was calibrated using standard Atto488 dye solutions to determine the structure factor (S) and confocal volume (V). Samples were placed on high-quality glass coverslips (CG15CH/CG15KH, Thorlabs, USA) and measured directly on the objective using immersion optics. Autocorrelation curves were recorded for 10 s per acquisition, with 10 independent measurements collected for each condition to ensure statistical robustness. Data were analyzed using Correlation Analysis software (LightEdge Technologies Limited, China). Diffusion coefficients and corresponding hydrodynamic radii were extracted from autocorrelation fits.

Data are presented as mean ± SD from 10 independent measurements. Statistical significance was evaluated using Two-sample t-tests, with each DDX21 concentration compared against the 0 μM control condition. Statistical analyses were performed using OriginPro. Significance levels are indicated as NS (not significant), **p < 0.01, and ****p < 0.0001.

**Stopped-Flow FRET Assay.** Real-time RNA duplex unwinding and strand annealing were monitored using a Bio-Logic SFM-400 stopped-flow instrument in a buffer containing 20 mM Tris–HCl (pH 7.5), 20 mM KCl, and 2 mM MgCl₂. Pre-annealed FAM/HEX-labeled RNA duplexes (4 nM) were rapidly mixed with 100 nM N protein, DDX21, or both proteins, in the presence or absence of 2 mM ATP. After rapid mixing, FAM fluorescence intensities were recorded over 16 seconds. Changes in FAM intensity were used to quantify duplex unwinding, with 100% unwinding calibrated using a mixture of two separate single-stranded RNAs. For annealing assays, complementary single-stranded RNAs (4 nM each), individually labeled with FAM or HEX, were mixed with proteins under identical conditions, and strand annealing was quantified by the decrease in FAM intensity over time, with 100% annealing calibrated using the pre-annealed duplex. All measurements were repeated at least five times, and the data were averaged for analysis.

**Table S1. Predicted PQSs on both positive and negative strands.**

| No. | Position | | Length | | | Sequences | QGRS Mapper score | | pqs finder score | | | G4 hunter score | |  |
| --- | --- | --- | --- | --- | --- | --- | --- | --- | --- | --- | --- | --- | --- | --- |
|  |  | | |  | | **Positive strand** |  | |  | |  |  |  |  |
| 1 | 353 | | | 25 | | GGCTTTGGAGACTCCGTGGAGGAGG | 16 | |  | |  |  |  |  |
| 2 | 359 | 19 | | | GGAGACTCCGTGGAGGAGG | | 13 | | 21 | |  |  |  |  |
| 3 | 370 | 20 | | | GGAGGAGGTCTTATCAGAGG | | 12 | |  | |  |  |  |  |
| 4 | 644 | 20 | | | GGTAATAAAGGAGCTGGTGG | | 15 | | 20 | |  |  |  |  |
| 5 | 653 | 23 | | | GGAGCTGGTGGCCATAGTTACGG | | 12 | |  | |  |  |  |  |
| 6 | 1463 | 26 | | | GGTGGTCGCACTATTGCCTTTGGAGG | | 6 | | 12 | |  |  |  |  |
| 7 | 1574 | 26 | | | GGTGTTGTTGGAGAAGGTTCCGAAGG | | 18 | | 12 | |  |  |  |  |
| 8 | 2714 | 29 | | | GGCGGTGCACCAACAAAGGTTACTTTTGG | | 10 | |  | |  |  |  |  |
| 9 | 3467 | 17 | | | GGAGGAGGTGTTGCAGG | | 15 | | 24 | |  |  |  |  |
| 10 | 3470 | 28 | | | GGAGGTGTTGCAGGAGCCTTAAATAAGG | | 10 | |  | |  |  |  |  |
| 11 | 4162 | 27 | | | GGTTATACCTACTAAAAAGGCTGGTGG | | 6 | | 11 | |  |  |  |  |
| 12 | 4262 | 28 | | | GGTTTAAATGGTTACACTGTAGAGGAGG | | 10 | | 10 | |  |  |  |  |
| 13 | 8687 | 23 | | | GGATACAAGGCTATTGATGGTGG | | 14 | | 16 | |  |  |  |  |
| 14 | 10255 | 28 | | | GGTACAGGCTGGTAATGTTCAACTCAGG | | 9 | | 10 | |  |  |  |  |
| 15 | 10261 | 30 | | | GGCTGGTAATGTTCAACTCAGGGTTATTGG | | 9 | |  | |  |  |  |  |
| 16 | 13385 | 20 | | | GGTATGTGGAAAGGTTATGG | | 19 | | 20 | |  |  |  |  |
| 17 | 14947 | 28 | | | GGTTTTCCATTTAATAAATGGGGTAAGG | | 4 | | 10 | |  |  |  |  |
| 18 | 15208 | 27 | | | GGAACAAGCAAATTCTATGGTGGTTGG | | 6 | | 11 | |  |  |  |  |
| 19 | 15448 | 29 | | | GGCGGTTCACTATATGTTAAACCAGGTGG | | 3 | |  | |  |  |  |  |
| 20 | 18296 | 23 | | | GGATTGGCTTCGATGTCGAGGGG | | 9 | | 16 | | 1 |  |  |  |
| 21 | 22316 | 29 | | | GGTGATTCTTCTTCAGGTTGGACAGCTGG | | 10 | |  | |  |  |  |  |
| 22 | 24200 | 29 | | | GGTACAATCACTTCTGGTTGGACCTTTGG | | 10 | |  | |  |  |  |  |
| 23 | 24215 | 20 | | | GGTTGGACCTTTGGTGCAGG | | 17 | | 20 | |  |  |  |  |
| 24 | 24268 | 24 | | | GGCTTATAGGTTTAATGGTATTGG | | 19 | | 15 | |  |  |  |  |
| 25 | 25197 | 22 | | | GGCCATGGTACATTTGGCTAGG | | 17 | | 17 | |  |  |  |  |
| 26 | 25203 | 28 | | | GGTACATTTGGCTAGGTTTTATAGCTGG | | 14 | |  | |  |  |  |  |
| 27 | 25951 | 29 | | | GGTGGTTATACTGAAAAATGGGAATCTGG | | 8 | |  | |  |  |  |  |
| 28 | 26746 | 30 | | | GGATCACCGGTGGAATTGCTATCGCAATGG | | 7 | |  | |  |  |  |  |
| 29 | 28781 | 29 | | | GGCTTCTACGCAGAAGGGAGCAGAGGCGG | | 9 | |  | |  |  |  |  |
| 30 | 28881 | 28 | | | GGGGAACTTCTCCTGCTAGAATGGCTGG | | 3 | | 27 | |  |  |  |  |
| 31 | 28903 | 15 | | | GGCTGGCAATGGCGG | | 18 | |  | |  |  |  |  |
| 32 | 29123 | 19 | | | GGAAATTTTGGGGACCAGG | | 14 | | 21 | |  |  |  |  |
| 33 | 29132 | 26 | | | GGGGACCAGGAACTAATCAGACAAGG | | 7 | |  | |  |  |  |  |
| 34 | 29234 | 30 | | | GGCATGGAAGTCACACCTTCGGGAACGTGG | | 11 | |  | |  |  |  |  |
|  |  |  | | |  | |  | |  | |  |  |  |  |
|  |  |  | | | **Negative strand** | |  | |  | |  |  |  |  |
| 1 | -29718 | 22 | | | GGCCTCGGTGAAAATGTGGTGG | | 13 | | 17 | |  |  |  |  |
| 2 | -26889 | 29 | | | | GGTCTGGTCAGAATAGTGCCATGGAGTGG | | 9 | |  | |  | |  |
| 3 | -23053 | 30 | | | | GGTTGGTAACCAACACCATTAGTGGGTTGG | | 6 | |  | |  | |  |
| 4 | -18457 | 28 | | | | GGTGTTTAAATTGATCTCCAGGCGGTGG | | 4 | |  | |  | |  |
| 5 | -18440 | 25 | | | | GGCGGTGGTTTAGCACTAACTCTGG | | 7 | | 14 | |  | |  |
| 6 | -16750 | 19 | | | | GGTTAAGTGGTGGTCTAGG | | 16 | | 21 | |  | |  |
| 7 | -15934 | 27 | | | | GGCCCCTAGGATTCTTGATGGATCTGG | | 16 | |  | |  | |  |
| 8 | -15924 | 29 | | | | GGATTCTTGATGGATCTGGGTAAGGAAGG | | 17 | |  | |  | |  |
| 9 | -15941 | 18 | | | | GGATCTGGGTAAGGAAGG | | 18 | | 23 | |  | |  |
| 10 | -13255 | 27 | | | | GGATTTGGATGATCTATGTGGCAACGG | | 14 | | 11 | |  | |  |
| 11 | -10039 | 25 | | | | GGTGATAGAGGTTTGTGGTGGTTGG | | 19 | |  | |  | |  |
| 12 | -10015 | 16 | | | | GGTTTGTGGTGGTTGG | | 17 | | 26 | |  | |  |
| 13 | -6011 | 17 | | | | GGATATGGTTGGTTTGG | | 19 | | 24 | |  | |  |
| 14 | -5996 | 26 | | | | GGTTGGTTTGGTACAAGATCAATTGG | | 10 | |  | |  | |  |
| 15 | -4886 | 16 | | | | GGTGGAATGTGGTAGG | | 17 | | 26 | |  | |  |
| 16 | -2449 | 24 | | | | GGGGCTTTTAGAGGCATGAGTAGG | | 13 | | 15 | | -1.04 | |  |
| 17 | -15 | 23 | | | | GGTTGGTTTGTTACCTGGGAAGG | | 13 | | 16 | |  | |  |
| 18 | -28289 | 25 | | | | GGGGTGCATTTCGCTGATTTTGGGG | |  | |  | | -1.28 | |  |

**Table S2. Nucleic acid sequences used in the study.**

|  | Number | Name | Sequence (5' to 3') |
| --- | --- | --- | --- |
|  | **Sequences for CD, CD-melting, fluorescent probe, and NMR assay^a^** | | |
|  | 1 | 353 | 5'-GGCUUUGGAGACUCCGUGGAGGAGG-3' |
|  | 2 | 359 | 5'-GGAGACUCCGUGGAGGAGG-3' |
|  | 3 | 370 | 5'-GGAGGAGGUCUUAUCAGAGG-3' |
|  | 4 | 644 | 5'-GGUAAUAAAGGAGCUGGUGG-3' |
|  | 5 | 1574 | 5'-GGUGUUGUUGGAGAAGGUUCCGAAGG-3' |
|  | 6 | 2714 | 5'-GGCGGUGCACCAACAAAGGUUACUUUUGG-3' |
|  | 7 | 3467 | 5'-GGAGGAGGUGUUGCAGG-3' |
|  | 8 | 4261 | 5'-GGGUUUAAAUGGUUACACUGUAGAGGAGG-3' |
|  | 9 | 8687 | 5'-GGAUACAAGGCUAUUGAUGGUGG-3' |
|  | 10 | 10261 | 5'-GGCUGGUAAUGUUCAACUCAGGGUUAUUGG-3' |
|  | 11 | 13385 | 5'-GGUAUGUGGAAAGGUUAUGG-3' |
|  | 12 | 18296 | 5'-GGAUUGGCUUCGAUGUCUAGGGG-3' |
|  | 13 | 24215 | 5'-GGUUGGACCUUUGGUGCAGG-3' |
|  | 14 | 24268 | 5'-GGCUUAUAGGUUUAAUGGUAUUGG-3' |
|  | 15 | 25197 | 5'-GGCCAUGGUACAUUUGGCUAGG-3' |
|  | 16 | 25951 | 5'-GGUGGUUAUACUGAAAAAUGGGAAUCUGG-3' |
|  | 17 | 26746 | 5'-GGAUCACCGGUGGAAUUGCUAUCGCAAUGG-3' |
|  | 18 | 28903 | 5'-GGCUGGCAAUGGCGG-3' |
|  | 19 | 29123 | 5'-GGAAAUUUUGGGGACCAGG-3' |
|  | 20 | -15 | 5'-GGUUGGUUUGUUACCUGGGAAGG-3' |
|  | 21 | -29718 | 5'-GGCCUCGGUGAAAAUGUGGUGG-3' |
|  | 22 | -4886 | 5'-GGUGGAAUGUGGUAGG-3' |
|  | 23 | -6011 | 5'-GGAUAUGGUUGGUUUGG-3' |
|  | 24 | -10015 | 5'-GGUUUGUGGUGGUUGG-3' |
|  | 25 | -10019 | 5'-GGUGAUAGAGGUUUGUGGUGG-3' |
|  | 26 | -16750 | 5'-GGUUAAGUGGUGGUCUAGG-3' |
|  | 27 | -15924 | 5'-GGAUCUGGGUAAGGAAGG-3' |
|  | 28 | -13255 | 5'-GGAUUUGGAUGAUCUAUGUGGCAACGG-3' |
|  | 29 | -10039 | 5'-GGUGAUAGAGGUUUGUGGUGGUUGG-3' |
|  | 30 | -2449 | 5'-GGGGCUUUUAGAGGCAUGAGUAGG-3' |
|  | 31 | -28289 | 5'-GGGGUGCAUUUCGCUGAUUUUGGGG-3' |
|  |  |  |  |
|  | **Control sequences for fluorescent probe assay** | | |
|  | ssRNA | | 5'-GCUCUGGUGCCGACCAACGAGAGU-3' |
|  | TERRA | | 5'-GGGUUAGGGUUAGGGUUAGGG-3' |
|  | |  |  |
| **Sequences for FRET-melting assay** | | | |
| F-359-T  F-1574-T  F-28903-T  F-25197-T  F-26746-T  F-28289-T  F-TERRA-T | | | FAM-GGAGACUCCGUGGAGGAGG-TAMRA  FAM-GGUGUUGUUGGAGAAGGUUCCGAAGG-TAMRA  FAM-GGCUGGCAAUGGCGG-TAMRA  FAM-GGCCAUGGUACAUUUGGCUAGG-TAMRA  FAM-GGAUCACCGGUGGAAUUGCUAUCGCAAUGG-TAMRA  FAM-GGGGUGCAUUUCGCUGAUUUUGGGG-TAMRA  FAM-GGGUUAGGGUUAGGGUUAGGG-TAMRA |
|  | | | |
| **Sequences for binding assay** | | | |
| FAM-359  FAM-1574  FAM-ssRNA  FAM-TERRA  FAM-TERRA-12U  FAM-12U-TERRA  FAM-12U-HP  FAM-HP-12U  FAM-ssDNA  FAM-G4-10T  FAM-G4  FAM-111  FAM-10T-111 | | | FAM-GGAGACUCCGUGGAGGAGG  FAM-GGUGUUGUUGGAGAAGGUUCCGAAGG |
|  |  |  | UUUUUUUUUUCUCUGCUCGACG-FAM |
|  |  |  | FAM-GGGUUAGGGUUAGGGUUAGGG |
|  |  |  | FAM-GGGUUAGGGUUAGGGUUAGGGUUUUUUUUUUUU |
|  |  |  | FAM-UUUUUUUUUUUUGGGUUAGGGUUAGGGUUAGGG  FAM-UUUUUUUUUUUUUCUCUGCUCGACGUUUUUUCGUCGAGCAGAG  FAM-UCUCUGCUCGACGUUUUUUCGUCGAGCAGAGUUUUUUUUUUUU  CACTGGCCGTCTTACGGTCGCTCTGCTCGACG-FAM  GGGTTAGGGTTAGGGTTAGGGTTTTTTTTTT-FAM  FAM-TGGGTTAGGGTTAGGGTTAGGG  FAM-GGGTGGGTGGGTGGG |
|  |  |  | FAM-TTTTTTTTTTGGGTGGGTGGGTGGG |
|  |  | **Sequence for gel filtration** | |
|  | 7T-111-7T | TTTTTTTGGGTGGGTGGGTGGGTTTTTTT | |
|  |  |  | |
|  |  | **Sequences of single-molecule FRET assay^b^** | |
|  | 359-Cy3 | CGUCGAGCAGAGUUUUGGAGACUCCGUGGAGGAGGU-Cy3 | |
|  | 359Mut-Cy3 | CGUCGAGCAGAGUUUUUUAUACUCCUUUUAUUAUUU-Cy3 | |
|  | 24U-Cy3 | CGUCGAGCAGAGUUUUUUUUUUUUUUUUUUUUUUUU-Cy3 | |
|  | 4U-Cy3 | CGUCGAGCAGAGUUUU-Cy3 | |
|  | 1574-Cy3 | CGUCGAGCAGAGUUUUGGUGUUGUUGGAGAAGGUUCCGAAGGU-Cy3 | |
|  | 12nt-Cy5 | Cy5-CTCTGCTCGACG-biotin | |
|  |  |  | |
|  |  | **Sequences for stopped-flow unwinding and annealing assay^c^** | |
|  | FA | HEX-CGUCGAGCAGAGUUUUUUUUUU | |
|  | FB | CUCUGCUCGACG-FAM | |
|  | FC | HEX-CGUCGAGCAGAG | |
|  | FD | UUUUUUUUUUCUCUGCUCGACG-FAM | |
|  |  |  | |
|  |  | **Sequences for cell transfection** | |
|  | Cy5-359 | Cy5-AGGAGACUCCGUGGAGGAGG | |
|  | Cy5-1574 | Cy5-AGGUGUUGUUGGAGAAGGUUCCGAAGG | |
|  | Cy5-28903 | Cy5-AGGCUGGCAAUGGCGG | |
|  |  |  | |

**a.** The DNA sequences used for CD measurements in Figure S3 were the DNA counterparts of the corresponding RNA sequences.

**b.** The Cy3- and Cy5-labeled strands assemble into a structure in which a 4-nt RNA linker connects the G4 motif to the duplex region.

**c.** Different combinations of RNA strands generate distinct duplex architectures: FA and FB form a 3′-overhanged RNA duplex; FA and FD form a forked RNA; FB and FC form a blunt-ended RNA duplex; and FC and FD form a 5′-overhanged RNA duplex.

**Table S3. Melting temperatures and van’t Hoff thermodynamic parameters of RNA G-Quadruplexes derived from CD melting analysis**

| Sequence | Enthalpy, ΔH (kJ/mol) | Melting Temperature, Tm (°C) | Melting Temperature, Tm (K) | Entropy,  ΔS (kJ/mol·K) | Free Energy at 25°C,  ΔG (kJ/mol) | Free Energy at 37°C,  ΔG (kJ/mol) |
| --- | --- | --- | --- | --- | --- | --- |
| 353 | 136.7 | 71.3 | 344.45 | 0.396865 | 18.3748 | 13.6125 |
| 359 | 111.5 | 76.4 | 349.55 | 0.318982 | 16.3957 | 12.5679 |
| 370 | 144.7 | 38.7 | 311.85 | 0.464005 | 6.3569 | 0.7888 |
| 644 | 151.3 | 40.7 | 313.85 | 0.482077 | 7.5686 | 1.7837 |
| 1574 | 118 | 54.4 | 327.55 | 0.36025 | 10.5914 | 6.2684 |
| 2714 | 133.9 | 54.9 | 328.05 | 0.408169 | 12.2043 | 7.3062 |
| 3467 | 230.4 | 48.6 | 321.75 | 0.716084 | 16.8996 | 8.3066 |
| 4261 | 126.6 | 39.6 | 312.75 | 0.404796 | 5.91 | 1.0525 |
| 8687 | 137.5 | 40.1 | 313.25 | 0.438947 | 6.6281 | 1.3607 |
| 10261 | 141.5 | 47.4 | 320.55 | 0.441429 | 9.888 | 4.5909 |
| 13385 | 153 | 36.6 | 309.75 | 0.493947 | 5.7298 | -0.1976 |
| 18296 | 128.9 | 55.5 | 328.65 | 0.392211 | 11.9624 | 7.2559 |
| 24215 | 146.1 | 56.8 | 329.95 | 0.442794 | 14.0809 | 8.7673 |
| 24268 | 140.4 | 35.9 | 309.05 | 0.454295 | 4.9518 | -0.4997 |
| 25197 | 128.9 | 67.2 | 340.35 | 0.378728 | 15.9823 | 11.4376 |
| 25951 | 121.1 | 47.2 | 320.35 | 0.378024 | 8.3921 | 3.8558 |
| 26746 | 124.4 | 57 | 330.15 | 0.376798 | 12.0575 | 7.536 |
| 28903 | 160.3 | 54 | 327.15 | 0.489989 | 14.2097 | 8.3298 |
| 29123 | 140.1 | 45.4 | 318.55 | 0.439805 | 8.972 | 3.6944 |
| -15 | 105.8 | 50.9 | 324.05 | 0.326493 | 8.4562 | 4.5383 |
| -29718 | 114.5 | 49.7 | 322.85 | 0.354654 | 8.76 | 4.5041 |
| -4886 | 197.9 | 43 | 316.15 | 0.625969 | 11.2674 | 3.7558 |
| -6011 | 215.7 | 37.5 | 310.65 | 0.694351 | 8.6794 | 0.3472 |
| -10015 | 266.5 | 47.8 | 320.95 | 0.830347 | 18.9319 | 8.9678 |
| -10019 | 178.1 | 44 | 317.15 | 0.561564 | 10.6697 | 3.9309 |
| -16750 | 189.6 | 39.9 | 313.05 | 0.605654 | 9.0242 | 1.7564 |
| -15924 | 136.8 | 44 | 317.15 | 0.431342 | 8.1955 | 3.0194 |
| -13255 | 128.7 | 46.2 | 319.35 | 0.403006 | 8.5437 | 3.7077 |
| -10039 | 198.9 | 48.2 | 321.35 | 0.618951 | 14.3597 | 6.9323 |
| -2449 | 133.7 | 52.9 | 326.05 | 0.41006 | 11.4407 | 6.52 |
| -28289 | 100.4 | 44.5 | 317.65 | 0.316071 | 6.1634 | 2.3705 |

**Table S4. Associations of sequence and loop lengths with Tm, ΔH, and ΔS.**

| **Length (nt)** | **Response variable** | **n** | **Spearmanρ** | **Spearman permutation p** | **Pearson r** | **Pearson permutation p** | **Direction** |
| --- | --- | --- | --- | --- | --- | --- | --- |
| Loop 1 length | Tm (°C) | 31 | -0.1323 | 0.4625 | 0.0138 | 0.9422 | negative |
| Loop 1 length | ΔH (kJ/mol) | 31 | 0.0376 | 0.8406 | 0.0237 | 0.8946 | positive |
| Loop 1 length | ΔS (J/mol·K) | 31 | 0.0399 | 0.8202 | 0.0281 | 0.8712 | positive |
| Loop 2 length | Tm (°C) | 31 | 0.1299 | 0.4817 | 0.0464 | 0.8146 | positive |
| Loop 2 length | ΔH (kJ/mol) | 31 | -0.5036 | 0.0032 | -0.5243 | 0.0030 | negative |
| Loop 2 length | ΔS (J/mol·K) | 31 | -0.4881 | 0.0070 | -0.5173 | 0.0024 | negative |
| Loop 3 length | Tm (°C) | 31 | 0.1221 | 0.5117 | 0.0785 | 0.6589 | positive |
| Loop 3 length | ΔH (kJ/mol) | 31 | 0.0910 | 0.6393 | -0.0351 | 0.8498 | positive |
| Loop 3 length | ΔS (J/mol·K) | 31 | 0.0966 | 0.6125 | -0.0438 | 0.8102 | positive |
| Total loop length | Tm (°C) | 31 | 0.1948 | 0.2925 | 0.1159 | 0.5401 | positive |
| Total loop length | ΔH (kJ/mol) | 31 | -0.5885 | 0.0010 | -0.5675 | 0.0004 | negative |
| Total loop length | ΔS (J/mol·K) | 31 | -0.5707 | 0.0010 | -0.5640 | 0.0014 | negative |
| Full sequence length | Tm (°C) | 31 | 0.1948 | 0.2891 | 0.1159 | 0.5287 | positive |
| Full sequence length | ΔH (kJ/mol) | 31 | -0.5885 | 0.0010 | -0.5675 | 0.0002 | negative |
| Full sequence length | ΔS (J/mol·K) | 31 | -0.5707 | 0.0012 | -0.5640 | 0.0004 | negative |

**Note.** Spearman ρ and Pearson r range from −1 to +1; their signs indicate the direction of association, whereas their absolute values indicate the strength of the association. Two-sided empirical permutation p values were calculated from 5,000 permutations, and p < 0.05 was considered nominally significant.

In the analyzed G₂-loop1-G₂-loop2-G₂-loop3-G₂ framework, total loop length is mathematically related to full sequence length (total loop length = sequence length − 8 nt). Thus, these two variables represent the same overall length effect and are not treated as independent predictors. Because ΔS was derived from ΔH and Tm rather than measured independently, associations of ΔH and ΔS with loop length should likewise be interpreted as linked thermodynamic relationships rather than independent evidence. None of the loop-length variables showed a significant association with Tm.

**Table S5. Loop features of the 31 experimentally tested PQSs.**

| No. | **Name** | **Tm (°C)** | **Stability** | **Loop 1 length** | **Loop 2 length** | **Loop 3 length** | **Loop 1 sequence** | **Loop 2 sequence** | **Loop 3 sequence** | **All-loop GC** | **All-loop AU** | **All-loop C** | **All-loop U** | **C-minus-U** | **Shannon entropy** |
| --- | --- | --- | --- | --- | --- | --- | --- | --- | --- | --- | --- | --- | --- | --- | --- |
| 1 | 353 | 71.3 | high | 4 | 9 | 4 | CUUU | AGACUCCGU | AGGA | 0.471 | 0.529 | 0.235 | 0.294 | -0.059 | 1.993 |
| 2 | 359 | 76.4 | high | 9 | 1 | 1 | AGACUCCGU | A | A | 0.455 | 0.545 | 0.273 | 0.182 | 0.091 | 1.936 |
| 3 | 370 | 38.7 | low | 1 | 1 | 10 | A | A | UCUUAUCAGA | 0.250 | 0.750 | 0.167 | 0.333 | -0.167 | 1.784 |
| 4 | 644 | 40.7 | low | 7 | 4 | 1 | UAAUAAA | AGCU | U | 0.167 | 0.833 | 0.083 | 0.333 | -0.250 | 1.626 |
| 5 | 1574 | 54.4 | moderate | 7 | 4 | 7 | UGUUGUU | AGAA | UUCCGAA | 0.333 | 0.667 | 0.111 | 0.389 | -0.278 | 1.878 |
| 6 | 2714 | 54.9 | moderate | 1 | 12 | 8 | C | UGCACCAACAAA | UUACUUUU | 0.333 | 0.667 | 0.286 | 0.333 | -0.048 | 1.782 |
| 7 | 3467 | 48.6 | low | 1 | 1 | 7 | A | A | UGUUGCA | 0.333 | 0.667 | 0.111 | 0.333 | -0.222 | 1.891 |
| 8 | 4261 | 39.6 | low | 8 | 12 | 1 | GUUUAAAU | UUACACUGUAGA | A | 0.238 | 0.762 | 0.095 | 0.381 | -0.286 | 1.785 |
| 9 | 8687 | 40.1 | low | 6 | 8 | 1 | AUACAA | CUAUUGAU | U | 0.200 | 0.800 | 0.133 | 0.400 | -0.267 | 1.706 |
| 10 | 10261 | 47.4 | low | 2 | 14 | 6 | CU | UAAUGUUCAACUCA | GUUAUU | 0.273 | 0.727 | 0.182 | 0.455 | -0.273 | 1.790 |
| 11 | 13385 | 36.6 | low | 5 | 3 | 4 | UAUGU | AAA | UUAU | 0.083 | 0.917 | 0 | 0.500 | -0.500 | 1.325 |
| 12 | 18296 | 55.5 | moderate | 3 | 12 | 0 | AUU | CUUCGAUGUCGA |  | 0.400 | 0.600 | 0.200 | 0.400 | -0.200 | 1.922 |
| 13 | 24215 | 56.8 | moderate | 2 | 6 | 4 | UU | ACCUUU | UGCA | 0.333 | 0.667 | 0.250 | 0.500 | -0.250 | 1.730 |
| 14 | 24268 | 35.9 | low | 6 | 6 | 4 | CUUAUA | UUUAAU | UAUU | 0.062 | 0.938 | 0.062 | 0.625 | -0.562 | 1.198 |
| 15 | 25197 | 67.2 | high | 4 | 7 | 3 | CCAU | UACAUUU | CUA | 0.286 | 0.714 | 0.286 | 0.429 | -0.143 | 1.557 |
| 16 | 25951 | 47.2 | low | 1 | 14 | 6 | U | UUAUACUGAAAAAU | GAAUCU | 0.190 | 0.810 | 0.095 | 0.381 | -0.286 | 1.700 |
| 17 | 26746 | 57 | moderate | 6 | 1 | 15 | AUCACC | U | AAUUGCUAUCGCAAU | 0.364 | 0.636 | 0.273 | 0.318 | -0.045 | 1.877 |
| 18 | 28903 | 54 | moderate | 2 | 4 | 1 | CU | CAAU | C | 0.429 | 0.571 | 0.429 | 0.286 | 0.143 | 1.557 |
| 19 | 29123 | 45.4 | low | 7 | 0 | 4 | AAAUUUU |  | ACCA | 0.182 | 0.818 | 0.182 | 0.364 | -0.182 | 1.495 |
| 20 | -15 | 50.9 | moderate | 2 | 10 | 3 | UU | UUUGUUACCU | GAA | 0.267 | 0.733 | 0.133 | 0.533 | -0.400 | 1.723 |
| 21 | -29718 | 49.7 | low | 4 | 9 | 1 | CCUC | UGAAAAUGU | U | 0.357 | 0.643 | 0.214 | 0.357 | -0.143 | 1.924 |
| 22 | -4886 | 43 | low | 1 | 5 | 2 | U | AAUGU | UA | 0.125 | 0.875 | 0 | 0.500 | -0.500 | 1.406 |
| 23 | -6011 | 37.5 | low | 4 | 2 | 3 | AUAU | UU | UUU | 0 | 1 | 0 | 0.778 | -0.778 | 0.764 |
| 24 | -10015 | 47.8 | low | 5 | 1 | 2 | UUUGU | U | UU | 0.125 | 0.875 | 0 | 0.875 | -0.875 | 0.544 |
| 25 | -10019 | 44 | low | 7 | 5 | 1 | UGAUAGA | UUUGU | U | 0.231 | 0.769 | 0 | 0.538 | -0.538 | 1.457 |
| 26 | -16750 | 39.9 | low | 6 | 1 | 4 | UUAAGU | U | UCUA | 0.182 | 0.818 | 0.091 | 0.545 | -0.455 | 1.617 |
| 27 | -15924 | 44 | low | 4 | 4 | 2 | AUCU | GUAA | AA | 0.200 | 0.800 | 0.100 | 0.300 | -0.200 | 1.685 |
| 28 | -13255 | 46.2 | low | 4 | 11 | 4 | AUUU | AUGAUCUAUGU | CAAC | 0.263 | 0.737 | 0.158 | 0.421 | -0.263 | 1.813 |
| 29 | -10039 | 48.2 | low | 7 | 5 | 5 | UGAUAGA | UUUGU | UGGUU | 0.294 | 0.706 | 0 | 0.529 | -0.529 | 1.447 |
| 30 | -2449 | 52.9 | moderate | 0 | 8 | 8 |  | CUUUUAGA | CAUGAGUA | 0.312 | 0.688 | 0.125 | 0.375 | -0.250 | 1.883 |
| 31 | -28289 | 44.5 | low | 0 | 17 | 0 |  | UGCAUUUCGCUGAUUUU |  | 0.353 | 0.647 | 0.176 | 0.529 | -0.353 | 1.732 |

**Table S6. Association between selected loop features and Tm.**

| **Loop feature** | **n** | **Spearman ρ** | **Spearman permutation p** | **Pearson r** | **Pearson permutation p** | **Direction** |
| --- | --- | --- | --- | --- | --- | --- |
| All-loop GC content | 31 | 0.823 | 0.000 | 0.778 | 0.000 | positive |
| All-loop AU content | 31 | -0.823 | 0.000 | -0.778 | 0.000 | negative |
| All-loop C content | 31 | 0.711 | 0.000 | 0.647 | 0.001 | positive |
| All-loop U content | 31 | -0.423 | 0.017 | -0.450 | 0.012 | negative |
| All-loop C-minus-U content | 31 | 0.624 | 0.000 | 0.592 | 0.000 | positive |
| All-loop Shannon entropy | 31 | 0.567 | 0.001 | 0.432 | 0.018 | positive |
| Loop 3 GC content | 29 | 0.525 | 0.003 | 0.362 | 0.050 | positive |
| Loop 3 AU content | 29 | -0.525 | 0.003 | -0.362 | 0.050 | negative |

**Table S7. Representative pairwise comparisons of overlapping or sequence-similar PQSs.**

| **Sequence A** | **Sequence B** | **Tm A (°C)** | **Tm B (°C)** | **Absolute ΔTm (°C)** | **Overlap (nt)** | **Sequence identity** | **Total loop length difference** | **All-loop C-content difference** | **All-loop U-content difference** | **Concatenated loops A** | **Concatenated loops B** |
| --- | --- | --- | --- | --- | --- | --- | --- | --- | --- | --- | --- |
| -10019 | -10039 | 44 | 48.2 | 4.200 | 21 | 0.840 | 4 | 0 | 0.009 | UGAUAGAUUUGUU | UGAUAGAUUUGUUGGUU |
| 353 | 359 | 71.3 | 76.4 | 5.100 | 19 | 0.760 | 6 | 0.037 | 0.112 | CUUUAGACUCCGUAGGA | AGACUCCGUAA |
| -10015 | -10039 | 47.8 | 48.2 | 0.400 | 16 | 0.640 | 9 | 0 | 0.346 | UUUGUUUU | UGAUAGAUUUGUUGGUU |
| 359 | 370 | 76.4 | 38.7 | 37.7 | 8 | 0.550 | 1 | 0.106 | 0.152 | AGACUCCGUAA | AAUCUUAUCAGA |
| 353 | 370 | 71.3 | 38.7 | 32.6 | 8 | 0.520 | 5 | 0.069 | 0.039 | CUUUAGACUCCGUAGGA | AAUCUUAUCAGA |
| 359 | 4261 | 76.4 | 39.6 | 36.8 | 0 | 0.552 | 10 | 0.177 | 0.199 | AGACUCCGUAA | GUUUAAAUUUACACUGUAGAA |
| 359 | 8687 | 76.4 | 40.1 | 36.3 | 0 | 0.565 | 4 | 0.139 | 0.218 | AGACUCCGUAA | AUACAACUAUUGAUU |
| 359 | -4886 | 76.4 | 43 | 33.4 | 0 | 0.579 | 3 | 0.273 | 0.318 | AGACUCCGUAA | UAAUGUUA |
| 359 | -15924 | 76.4 | 44 | 32.4 | 0 | 0.632 | 1 | 0.173 | 0.118 | AGACUCCGUAA | AUCUGUAAAA |
| 353 | 4261 | 71.3 | 39.6 | 31.7 | 0 | 0.655 | 4 | 0.140 | 0.087 | CUUUAGACUCCGUAGGA | GUUUAAAUUUACACUGUAGAA |
| 353 | -16750 | 71.3 | 39.9 | 31.4 | 0 | 0.560 | 6 | 0.144 | 0.251 | CUUUAGACUCCGUAGGA | UUAAGUUUCUA |
| 24268 | 25197 | 35.9 | 67.2 | 31.3 | 0 | 0.583 | 2 | 0.223 | 0.196 | CUUAUAUUUAAUUAUU | CCAUUACAUUUCUA |

**Figure S1. Sequence characteristics of the predicted PQSs**. **(A)** Length distribution of the predicted PQSs on both positive and negative strands. **(B)** Distribution of loop lengths within the predicted PQSs, based on the general motif G₂L₁G₂L₂G₂L₃G₂.

**Figure S2.** **CD spectra of the 31 selected PQSs.** Each RNA sequence was dissolved in 150 mM KCl at 4 µM, heated to 95°C, and slowly cooled to room temperature over 7 hours to facilitate G4 folding. Most spectra exhibit a positive peak at ~265 nm and a negative valley at 240 nm, characteristic of parallel G4 structures. In contrast, -10019 adopts a hybrid parallel/antiparallel conformation with two positive peaks (black arrow). The red arrow highlights an atypical spectrum for 24215, showing a peak at 258 nm.

**Figure S3. CD spectra of corresponding DNA sequences containing the general motif G₂L₁G₂L₂G₂L₃G₂.** Samples were prepared in 150 mM KCl at 4 µM, heated to 95°C, and slowly cooled to room temperature over 7 hours to facilitate G4 folding. **(A)** Most sequences lack G4-forming capability, as indicated by the absence of G4-specific CD signatures. **(B)** Only five sequences exhibit characteristic G4 CD spectra.

**Figure S4. Characterization of G4 formation by PQSs using the ThT probe in 150 mM KCl.** ThT concentration was maintained at 0.5 µM, while RNA concentrations ranged from 0 to 1 µM. **(A)** Positive control (TERRA) shows a significant increase in ThT fluorescence intensity with rising RNA concentration, whereas a 24-nt ssRNA (negative control) exhibits no change, confirming ThT as a sensitive G4 probe. **(B)** Most PQSs on the positive strand induce ThT emission increases with concentration, suggesting G4 formation. In contrast, 24215 shows no change, indicating it may not form a G4 structure. **(C)** All tested PQSs on the negative strand exhibit concentration-dependent ThT emission increases, consistent with G4 formation.

**Figure S5. G4 formation analysis of PQSs using the QUMA-1 probe in 150 mM KCl.** QUMA-1 was used at a fixed concentration of 0.5 µM, while RNA concentrations ranged from 0 to 1 µM. TERRA (positive control) exhibited a strong fluorescence increase with rising RNA concentration, whereas a 24-nt ssRNA (negative control) showed no change, confirming QUMA-1 as a sensitive G4 probe. All tested PQSs displayed significant fluorescence enhancement with increasing concentration, indicating G4 structure formation.

**Figure S6. CD-melting spectra of the PQSs in 150 mM KCl.** Each RNA sequence (4 μM) was dissolved in 150 mM KCl, heated to 95°C, and slowly cooled to room temperature over 7 hours to facilitate proper G4 folding. Melting curves were recorded from 25°C to 95°C.

**Figure S7. 1D ¹H NMR spectrum of 359-G4 in 150 mM KCl.** The peaks between 10.5–11.0 ppm correspond to imino protons involved in Hoogsteen hydrogen bonds, confirming G4 formation.

**Figure S8. FRET-melting curves of viral G4s in the presence of 2 μM G4 ligands.** Experiments were conducted with 0.5 μM G4 RNA in 25 mM Tris-HCl (pH 7.5) and 150 mM KCl. "Control" indicates samples without ligand addition.

**Figure S9.** **CD and CD-melting profiles of 4 selective PQSs in 150 mM KCl, NaCl, or LiCl**. The spectra show minimal dependence on the monovalent cation type.

 **Figure S10. CD spectra of 1574 and 28903-G4s pre-folded in 20 mM KCl in the presence of increasing [Mg²⁺] (0–2 mM).** No significant changes in CD signatures were observed, indicating that Mg²⁺ does not induce detectable alterations in G4 topology or overall folding.

**Figure S11. Effect of Ca²⁺ on the thermostability of SARS-CoV-2 G4.** Ca²⁺ produced a concentration-dependent stabilizing effect on 25197-G4 in the presence of 20 mM KCl. Mn²⁺ and Zn²⁺ were also tested under the same conditions but produced non-canonical fluorescence profiles without resolvable melting transitions, precluding reliable determination of Tm.

**Figure S12. Control experiments validating that the smFRET signals of 359-G4 reflect intrinsic G4 folding. (A)** FRET histograms and **(B)** representative single-molecule FRET trajectories of the 4-nt linker construct under 20, 50, 100, and 150 mM KCl, showing a stable high-FRET population (~0.95) with no detectable dynamic transitions. **(C)** FRET histograms under 150 mM KCl for 359-G4, its G-to-U mutant (359Mut), and poly-U controls. The 359Mut and length-matched poly-U (24-nt) exhibit broadened and lower-FRET distributions compared to 359-G4, whereas the 4-nt linker retains a high-FRET state similar to 359-G4, indicating that the observed high-FRET state originates from a compact, sequence-dependent G4 structure rather than duplex or nonspecific structural contributions.

**Figure S13.** **Representative FRET traces of 1574-G4. (A)** Representative slow-dynamic FRET traces showing transitions between distinct FRET states. **(B)** Dwell-time distributions for individual FRET states derived from HMM-idealized trajectories. The solid curves represent single-exponential fits, yielding the corresponding dwell-time constants (τ). **(C)** Representative fast-dynamic FRET traces showing rapid FRET fluctuations without reliably resolved dwell states.

**Figure S14. Mg²⁺-dependent conformational dynamics of the 1574-G4.** **(A)** Representative smFRET traces illustrating the three characteristic dynamic behaviors exhibited by 1574-G4. **(B)** Quantification of the relative proportions of the three trace classes across different KCl concentrations.

**Figure S15. Interactions of viral and host proteins with RNA G4s and nucleic acid substrates. (A)** Binding affinities of 359-G4 with various host and viral proteins measured by fluorescence anisotropy in 20, 50, and 100 mM KCl. Asterisks (*) indicate conditions in which K_D_ values were too weak to be reliably determined. **(B)** Binding affinities of SARS-CoV-2 N protein toward different nucleic acid constructs determined by fluorescence anisotropy. **(C)** Oligomeric states of N protein in the absence and presence of parallel G4 analyzed by size-exclusion chromatography, showing G4-dependent changes in assembly behavior. **(D)** Elution profile of the protein molecular weight standard mixture. **(E)** Calibration curve plotting the distribution coefficient ($K_{av}$) against the logarithm of molecular weight (MW). $K_{av}$ was calculated from the elution volume ($V_{e}$) using the equation $K_{av}=\left( V_{e}-V_{0} \right)/\left( V_{t}-V_{0} \right)$, where $V_{0}$ and $V_{t}$ are the void and total column volumes, respectively. The apparent MW of the N–G4 complexes was subsequently determined by interpolating their observed $K_{av}$ values into this standard calibration curve. **(F)** Quantification of static and dynamic smFRET trajectories of 1574-G4 in the presence of increasing concentrations of N protein (10–500 nM), indicating modulation of conformational dynamics.

**Figure S16. Cellular colocalization and specificity controls of N protein and 28903-G4.** The mCherry-tagged N protein is pseudocolored yellow, Cy5-labeled RNA is shown in red, and nuclei stained with Hoechst are shown in blue. **(A)** Single-transfection controls to rule out spectral bleed-through: cells were transfected with either Cy5-labeled 28903 RNA alone or N-mcherry alone. In each condition, only the corresponding introduced molecule was detected, together with Hoechst nuclear staining, confirming the specificity of each fluorescence channel. **(B)** Representative fluorescence images showing the distribution of N-mCherry, Cy5-labeled 28903, Hoechst-stained nuclei, and the merged channels. The line-scan profile on the right was generated along the white line indicated in the merged image and shows overlapping fluorescence intensity peaks of N-mcherry and Cy5-28903 RNA, supporting their spatial colocalization within cells. Scale bars, 10 µm.

**Figure S17. Competition binding assays between PhenDC3, TMPyP4, and N protein on the 359-G4. (A)** Binding curves and K_D_ values of PhenDC3 and N protein to the 359-G4. **(B)** Fluorescence anisotropy competition assay showing N protein displacing PhenDC3 from the 359-G4. Pre-folded 5 nM 359-G4 was incubated with varying concentrations of PhenDC3 at 37°C for 30 minutes, followed by the addition of increasing concentrations of N protein. **(C)** Binding curves and K_D_ values of TMPyP4 and N protein to the 359-G4. **(D)** Fluorescence anisotropy competition assay showing N protein displacing TMPyP4 from the 359-G4. Pre-folded 5 nM 359-G4 was incubated with varying concentrations of TMPyP4 at 37°C for 30 minutes, followed by the addition of increasing concentrations of N protein. Final anisotropy values stabilized at the level corresponding to N protein binding. Measurements were performed in 20 mM Tris-HCl (pH 7.5) and 20 mM KCl.

**Figure S18. Combined effects of DDX21 and the N protein on SARS-CoV-2 RNA G4 conformational remodeling. (A)** FRET distributions and representative traces of the 359-G4 in the absence and presence of 100 nM DDX3X with 2 mM ATP. **(B)** FRET distributions of the 1574-G4 in the absence and presence of 100 nM DDX21, with or without 2 mM ATP. **(C)** Apparent equilibrium dissociation constants (K_D_) of DDX21 for 359-G4 and 1574-G4 measured by fluorescence anisotropy in 20 mM Tris-HCl (pH 7.5) and 20 mM KCl, in the absence or presence of 2 mM Mg²⁺ or 2 mM ATP–Mg²⁺. Under all tested conditions, DDX21 exhibits high affinity toward both substrates. **(D)** Comparison of smFRET distributions of 359-G4 and 1574-G4 in the presence of DDX21 and ATP–Mg²⁺ with original 1574-G4. Notably, the FRET distribution of DDX21-remodeled 359-G4 closely resembles that of native 1574-G4. **(E)** FRET efficiency distributions of the 1574-G4 construct in the presence of 100 nM DDX21, 100 nM N protein, with or without 2 mM ATP.

**Figure S19. FCS control for RNA-independent N–DDX21 interaction.** Autocorrelation curves of Atto488-labeled N protein (20 nM) with or without a 10-fold excess of unlabeled 359-G4 RNA (200 nM) in the presence of 12 µM DDX21. The presence of RNA did not alter the DDX21-induced rightward shift of the autocorrelation curves, indicating that RNA bridging does not mediate the N–DDX21 interaction. Measurements were performed in 20 mM Tris-HCl (pH 7.5), 150 mM KCl.

**Figure S20. Stop-flow characterization of duplex RNA unwinding and strand annealing activities of N protein, DDX21, and the N–DDX21 combination.** Duplex RNA unwinding assays were performed using pre-annealed double-stranded RNA substrates, whereas strand annealing assays employed two complementary single-stranded RNAs capable of forming duplex RNA. All reactions were carried out with 4 nM RNA substrates and 100 nM protein, in the presence or absence of 2 mM ATP, as indicated. The reaction buffer contained 20 mM Tris–HCl (pH 7.5), 20 mM KCl, and 2 mM MgCl₂. **(A–B)** Unwinding and annealing activities of N protein. **(C–D)** Unwinding and annealing activities of DDX21. **(E)** Duplex RNA unwinding in the presence of both N protein and DDX21 in the absence of ATP. **(F)** Duplex RNA unwinding in the presence of both N protein and DDX21 with ATP. **(G)** Strand annealing activity of the N–DDX21 combination in the absence of ATP.

**Figure S21. Potential coexistence of G4s and alternative RNA structures. (A)** Secondary structure prediction of the 1574-G4-containing sequence generated using RNAfold suggests the formation of a local hairpin structure. **(B)** The 359-G4-forming sequence (indicated by the arrows) has previously been reported to reside within the SL7 stem–loop structure. Combined with our observation that this sequence can also adopt a highly stable G4 conformation, these findings suggest that the same RNA element may access different structural states depending on environmental conditions. Guanines highlighted in red indicate residues that can participate in G-tetrad formation within the G4 structure.
